# Microglial Activation and Inflammatory Responses in Parkinson's Disease Models Are Attenuated by TRPM2 Depletion

**DOI:** 10.1002/glia.70055

**Published:** 2025-07-15

**Authors:** Ana Flávia F. Ferreira, Zhong‐Ping Feng, Hong‐Shuo Sun, Luiz Roberto G. Britto

**Affiliations:** ^1^ Department of Physiology and Biophysics Institute of Biomedical Sciences, University of São Paulo São Paulo Brazil; ^2^ Department of Physiology Temerty Faculty of Medicine, University of Toronto Toronto Canada; ^3^ Department of Surgery Temerty Faculty of Medicine, University of Toronto Toronto Canada

**Keywords:** 6‐hydroxydopamine, CD68, co‐culture, cytokines, microglia, morphology, TRPM2 knockout

## Abstract

Inflammation, and particularly microglial cells, has become a central feature in Parkinson's disease (PD) pathology. The transient receptor potential melastatin 2 (TRPM2) is a calcium‐permeable nonselective channel involved in the pathological mechanism of several inflammatory and neurodegenerative diseases. However, the role of TRPM2 in inflammation and microglial activation in the context of PD remains unclear. Here, we combined both in vivo and in vitro PD models to investigate that question. Male and female TRPM2 partial and complete knockout mice were submitted to the 6‐hydroxidopamine mouse model of PD. We assessed microglia and lysosome‐associated protein (CD68) density levels, microglial morphology and cluster classification, CD68 area in individual microglial cells, and the protein levels of six different cytokines in the substantia nigra pars compacta and the striatum. Our results indicate that TRPM2 deletion reduced microglial density, rescued its morphology, decreased CD68 staining area within microglia, and lowered pro‐inflammatory cytokines levels in both male and female mice. To better understand TRPM2 involvement in PD pathology, we selectively knocked‐down TRPM2 in neurons, microglia, or both cells in a human neuron–microglia co‐culture PD model. An improvement in cell viability and a decrease in cell death were observed across the different experimental approaches. Lastly, TRPM2 deletion revealed reduced microglial phagocytosis and decreased expression of inflammation‐related molecules. For the first time, we demonstrated that TRPM2 is a critical mediator of microglial function in the context of PD. Thus, this study suggests that TRPM2 inhibition may offer a novel therapeutic target for PD modification.

## Main Points

- *Trpm2* partial and complete knockout reduce neuroinflammation in male and female mice.
- Microglial morphological changes are not found in *Trpm2* knockout 6‐OHDA‐induced mice.
- TRPM2 is involved in microglial phagocytosis and IL1β, IL6, and iNOS expression.

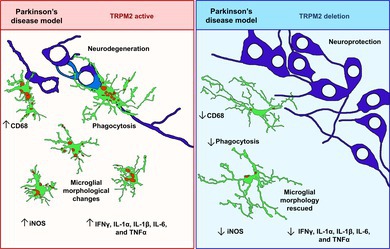

## 1 Introduction

Over the past few decades, microglia have garnered significant attention in neuroscience research due to their critical roles in the central nervous system (CNS). These yolk‐sac‐derived cells are essential for maintaining CNS homeostasis and facilitating processes such as tissue repair, neurogenesis, synapse formation and remodeling, inflammation, and myelination (Paolicelli and Ferretti 2017). As the main immune cells in the CNS, microglia keep brain homeostasis by phagocytizing misfolded proteins, debris, and dying cells while monitoring and protecting neuronal function (Cserép et al. 2020; Li and Barres 2018). These complex and dynamic cells can respond to changes in the environment, such as in a disease scenario, altering their morphology and molecular profile. Microglia are continuously active cells that assume different shapes and functions according to sex, age, brain region, and health state (Gao et al. 2023; Paolicelli et al. 2022; Vidal‐Itriago et al. 2022).

In neurodegenerative diseases, microglia are chronically activated and, consequently, can become detrimental, contributing to disease progression. Parkinson's disease (PD), the second most prevalent neurodegenerative disease, is characterized by motor impairments such as bradykinesia, rigidity, resting tremors, and postural instability (Balestrino and Schapira 2020). Its pathology is defined by the loss of dopaminergic neurons in the substantia nigra pars compacta (SNc), reduced dopamine release in the striatum (caudate‐putamen, CPu), and the accumulation of α‐synuclein that aggregates into Lewy bodies. Besides that, microglial activation, first described in PD patients in 1988 (McGeer et al. 1988), has become a well‐established hallmark of PD. The upregulation of inflammatory molecules, such as the inducible nitric oxide synthase (iNOS) (Hunot et al. 1996); the increase of the lysosome‐associated protein CD68, a phagocytosis‐associated marker (Doorn et al. 2014); and elevated levels of pro‐inflammatory cytokines (Nagatsu et al. 2000) were all observed in postmortem brains of PD patients. In light of that, strategies that target microglial function and inflammation emerge as potential therapeutic avenues for PD.

The transient receptor potential melastatin 2 (TRPM2) is a calcium‐permeable nonselective channel also known as an oxidative stress‐activated channel (Fonfria et al. 2004). TRPM2 is ubiquitously expressed and is the most abundant TRP in the brain, where it is found in neurons, astrocytes, and microglia (Bond and Greenfield 2007; Kaneko et al. 2006; Kraft et al. 2004; Lee et al. 2010). This channel plays a crucial role in calcium signaling and the regulation of cellular calcium homeostasis. However, its dysfunction has been implicated in a variety of pathological conditions, including several neurological and neurodegenerative diseases (Maliougina and El Hiani 2023). Studies have demonstrated that TRPM2 contributes to neurodegeneration and inflammatory processes in Alzheimer's disease, ischemia, multiple sclerosis, and epilepsy (Huang et al. 2017; Jiang et al. 2018; Shao et al. 2021; Tsutsui et al. 2018; Zong et al. 2022), suggesting TRPM2 as a promising therapeutic target.

Previous studies by our group and others have demonstrated that TRPM2 pharmacological inhibition is neuroprotective in PD animal models (Ferreira et al. 2022; Ferreira, Ulrich, Feng, et al. 2024a; Tamura et al. 2022; Vaidya et al. 2021). However, the use of TRPM2 inhibitors poses a clear limitation, as no selective inhibitor is currently available (Zhang et al. 2020). The use of TRPM2 knockout has shown alleviation of behavior impairment, neuronal death, and inflammation in other disease models (Huang et al. 2017; Miyanohara et al. 2018; Shao et al. 2021; Tsutsui et al. 2018). Only two studies have investigated the role of TRPM2 in PD by using TRPM2 knockout mice (Ferreira, Ulrich, Mori, et al. 2024b; Ye et al. 2023), yet they only demonstrated prevention of dopaminergic neuronal death and motor deficits. Therefore, the role of the TRPM2 channel in PD, especially its involvement in inflammation and microglial activation, remains poorly explored. In this study, we aimed to investigate the TRPM2 contribution to microglial function and inflammatory responses in PD. To address this, we submitted TRPM2 partial and complete knockout male and female mice to the 6‐hydroxidopamine (6‐OHDA) mouse model of PD and performed molecular and morphological profiling of microglia. Additionally, we carried out in vitro experiments in a human neuron–microglia co‐culture model of PD to evaluate the impact of TRPM2 ablation. Lastly, we assessed TRPM2 involvement in microglial phagocytosis and the expression of inflammatory molecules. To the best of our knowledge, this is the first study that uncovered TRPM2 as a critical mediator of microglial function in the context of PD.

## 2 Material and Methods

### 2.1 Animals

The TRPM2 knockout mice were kindly provided by Dr. Yasuo Mori (Yamamoto et al. 2008), and heterozygote animals (TRPM2+/−) were bred to obtain the genotypes used in this study. Genotyping was routinely performed as previously described (Ferreira, Ulrich, Mori, et al. 2024b). Thirty‐six adult male and 36 female 3‐month‐old mice were used. The mice were housed in a temperature‐controlled room with a 12‐h light/dark cycle and provided with a standard chow diet and water ad libitum. All procedures were conducted in accordance with the ARRIVE guidelines, the National Institutes of Health Guide for the Care and Use of Laboratory Animals (NIH Publications No. 8023, revised 1978), the guidelines of the National Council for the Control of Animal Experimentation (CONCEA, Brazil), and the Canadian Council on Animal Care guidelines. The study was approved by the Ethics Committee for Animal Research of the Institute of Biomedical Sciences of the University of São Paulo (CEUA‐ICB/USP, Brazil, #8395080450) and by the University of Toronto Animal Care Committee (Canada, AUP #20011561).

### 2.2 Stereotaxic Surgery

To generate the PD model, as previously described, 6‐hydroxidopamine (6‐OHDA) was injected into the right striatum (caudate‐putamen, CPu), as it is unable to cross the blood–brain barrier (Ferreira et al. 2022). Mice were deeply anesthetized with isoflurane mixed with oxygen (5% for induction and 2% for maintenance) and placed in the stereotaxic frame. Ten micrograms of 6‐OHDA (volume of 1 μL, H4381, Sigma‐Aldrich) diluted in 0.3% ascorbic acid in saline (vehicle, here called as saline) was administered to each animal at the following coordinates: anteroposterior (AP) − 0.4 mm; lateral (L) − 2.0 mm; and ventral (V) − 3.0 mm, using the bregma as a reference point. The injection was performed with a Hamilton syringe (Neuros Syringes—65,460–02) at a rate of 0.5 μL/min, and an extra 5 min was counted before the needle removal. Animals were observed until fully recovered. The postsurgical follow‐up care included daily observations, weight monitoring, and pain management with meloxicam (2 mg/kg, s.c) for 3 days. For female animals, the estrous cycles were tracked using vaginal lavage, and all stereotaxic surgeries were performed during the diestrus phase. Female and male animals were divided into six experimental groups: wild‐type animals that received saline injection (TRPM2+/+); wild‐type animals that received 6‐OHDA injection (TRPM2+/+ PD); heterozygote animals that received saline injection (TRPM2+/−); heterozygote animals that received 6‐OHDA injection (TRPM2+/− PD); homozygote animals that received saline injection (TRPM2−/−); and homozygote animals that received 6‐OHDA injection (TRPM2−/− PD). Mice were euthanized 7 days after the surgery for the analysis.

### 2.3 Immunofluorescence

Animals (*n* = 3/group) were transcardially perfused with 0.9% saline solution followed by 4% paraformaldehyde (PFA). The brain was dissected out, immersed in PFA for 4 h at 4°C, followed by immersion in 30% sucrose solution in 0.1 M phosphate buffer (PB, pH 7.4) for cryoprotection. A sliding freezing microtome (Leica SM2000 R, RRID:SCR_018456) was used to section the brains at 30 μm. Slides were incubated overnight with anti‐tyrosine hydroxylase (1:500; TH; Millipore Cat# MAB5280, RRID:AB_2201526), anti‐ionized calcium‐binding adaptor molecule 1 (1:1000; Iba1; FUJIFILM Wako Pure Chemical Corporation Cat# 019–19,741, RRID:AB_839504), and anti‐CD68 (1:500; Bio‐Rad Cat# MCA1957GA, RRID:AB_324217). Sections were then incubated for 2 h with anti‐mouse, rat, and rabbit secondary antibodies, Alexa Fluor 405, 488, and 568. Tissue was mounted on glass slides, covered with ProLong Gold Antifade Mountant (Invitrogen, P36930) and coverslipped.

### 2.4 Image Acquisition, Processing, and Analysis

Images were captured in a confocal laser scanning microscope (LSM700 Laser Scanning Microscope, Zeiss, Germany) with 20× and 40× objectives. Three to five coronal slices per animal were scanned. Z‐stack images were acquired using the ZEISS ZEN Microscopy Software (RRID:SCR_013672) and the maximal intensity projection was applied to generate high‐resolution images of the cells and their ramifications. Laser settings were kept consistent across all image acquisitions. Images from the SNc and the CPu were analyzed with Fiji free software (Fiji, RRID:SCR_002285) as previously described (Ferreira, Ulrich, Feng, et al. 2024a). In brief, 20× images were analyzed for the number of TH‐positive cells in the SNc (using the Cell Counter plugin) and the relative integrated density of Iba1 and CD68 in both the SNc and the CPu (limited by the Threshold plugin). For this analysis, data were normalized to the respective control groups (TRPM2+/+).

The 40× images were analyzed to evaluate the microglial morphology using the AnalyzeSkeleton(2D/3D) plugin and the FracLac toolbar as detailed in (Fernández‐ Arjona et al. 2017; Ferreira, Ulrich, Feng, et al. 2024a; León‐Rodríguez et al. 2025). In brief, (1) channel colors were split and the green channel (Iba1 staining) was used for the next steps; (2) contrast and brightness were adjusted; (3) a binary image was obtained and (4) skeletonized with the plugin AnalyzeSkeleton(2D/3D) to obtain the summed number of endpoints and the summed branch length, later divided by the number of microglial somas in the corresponding image. The binary image (Black and white) was (5) manually edited to isolate individual cells, fill gaps in the branches and cell body, and erase debris and other cells, always using the original image as reference. The outline shape was then processed in FracLac where 19 parameters were extracted (parameters can be consulted in Table S2). For the Fractal analysis, cells with a complete cell body and branches, and no overlap with neighboring cells, were selected. Between 15 and 25 cells were selected per group. For the SNc, the following number of cells were analyzed: male animals: TRPM2+/+ 20 cells; TRPM2+/+ PD 25 cells; TRPM2+/− 20 cells; TRPM2+/− PD 23 cells; TRPM2−/− 21 cells; TRPM2−/− PD 25 cells; female: TRPM2+/+ 15 cells; TRPM2+/+ PD 25 cells; TRPM2+/− 16 cells; TRPM2+/− PD 23 cells; TRPM2−/− 19 cells; TRPM2−/− PD 19 cells. For the CPu, the following number of cells were used: male animals: TRPM2+/+ 20 cells; TRPM2+/+ PD 20 cells; TRPM2+/− 19 cells; TRPM2+/− PD 20 cells; TRPM2−/− 20 cells; TRPM2−/− PD 19 cells; female: TRPM2+/+ 16 cells; TRPM2+/+ PD 20 cells; TRPM2+/− 16 cells; TRPM2+/− PD 18 cells; TRPM2−/− 22 cells; TRPM2−/− PD 21 cells.

We also assessed the area of CD68 staining within each Iba1‐positive cell. CD68 is a lysosomal marker shown to be more expressed in activated microglia (Chistiakov et al. 2017; Hwang et al. 2022). To calculate the percentage of CD68‐positive area per cell, we used the cell outlines generated from the previous Iba1 analysis to create regions of interest (ROIs) with the Create Selection tool in ImageJ. The red channel images (CD68 staining) were opened in ImageJ and the Threshold plugin was applied. The ROIs were then overlaid onto the CD68 channel and the area fraction was measured (the percentage of CD68‐positive pixels within each cell). All images were calibrated using the Set Scale tool. This method enabled cell‐by‐cell quantification of CD68 area/expression.

### 2.5 Enzyme‐Linked Immunosorbent Assay (ELISA)

Six cytokines (IFNγ, IL‐1α, IL‐1β, IL‐6, IL‐10, and TNFα) were measured in the midbrain and in the CPu (*n* = 3/group) using the Q‐Plex Mouse Cytokine Panel 1 HS (6‐Plex) (Cat#: 111349MS, Quansys Biosciences) and according to the manufacturer instructions. The tissue was homogenized in RIPA buffer (2 M NaCl; 1 M Tris, pH 7.4; 0.5 M EDTA; 10% Triton X‐100 (v/v); 10% sodium deoxycholate (w/v); 10% SDS (w/v); 1 M dithiothreitol) and centrifuged at 12,000 rpm twice. The Bradford method was used to determine protein levels. The calibrator curve and samples (20 μL/well in duplicate) were added to the plate and incubated for 3 h. After three washes, the plate was incubated with detection mix for 2 h. After three more washes, streptavidin HRP was incubated for 20 min. Finally, the plate was washed six times and the chemiluminescent substrate was added. The plate was immediately imaged on G:BOX Chemi XRQ and analyzed on Q‐View Software. Cytokine levels were normalized to the total protein content in each sample (pg/mg) (Bianchetti et al. 2024).

### 2.6 Cell Lines and Treatments

The human SH‐SY5Y neuroblastoma cells and the human microglial cell line (HMC3—CRL‐3304) were used. SH‐SY5Y were grown in Dulbecco's Modified Eagle's Medium: Nutrient Mixture F‐12 media (DMEM/F12, 11,330,032) supplemented with 10% fetal bovine serum (Gibco, 12,483,020), 100 units/mL penicillin, and 100 μg/mL streptomycin (Sigma, P4333) in a humidified incubator at 37°C with 5% CO_2_. Media were replaced every 3–5 days and cells were passed when they reached 90%–100% confluent. Dopaminergic neuron‐phenotype differentiation was induced by incubation with 10 μM of retinoic acid (RA, Cell Signaling Technology, 38255S) for 3 days followed by 80 nM of 12‐o‐tetradecanoylphorbol‐13‐acetate (TPA, BioShop, PMA168.1) for an additional 3 days (Presgraves et al. 2003) (Figure S1a). RA and TPA were diluted in DMSO (the final concentration did not exceed 0.1%) and added to Neurobasal medium (Gibco, 21,103–049) containing 2% B27 (Gibco, 17,504,044), 100 units/mL penicillin, and 100 μg/mL streptomycin (P/S, Sigma, P4333), and 2 mM GlutaMAX (Gibco, 35,050,061). Media were partially changed after 48 h and completely replaced between RA and TPA. HMC3 were grown in Eagle's minimum essential medium (EMEM, Cedarlane, 30–2003) supplemented with 10% fetal bovine serum, heat inactivated (hiFBS, Gibco, 12,484,028) and P/S. For assays, media were changed to EMEM with 1% hiFBS. When in co‐culture, the media used were neurobasal medium supplemented with B27, GlutaMAX, and P/S and cells were plated in a 1:3 ratio of neurons to microglia, to mimic the neuroinflammatory condition. Cells were used until passage 15. The PD model was induced by 6‐OHDA (diluted in sterile distilled water with 0.3% ascorbic acid, BioShop, ASO704.100). The toxin was added to the culture at varying concentrations to determine the dose that promoted around 60%–50% cell death. Treatment with 25 μM for 24 h was the selected one and thus it was used for subsequent experiments (Figure S1b–g). To induce TRPM2 knockdown, TRPM2‐specific siRNA (siRNA TRPM2 assay #S14425, Thermo Fisher, 4,392,420) or scramble control siRNA (Select Negative Control #1 siRNA) were transfected into cells using Lipofectamine RNAiMAX Transfection Reagent (Invitrogen, 13,778,075) and Opti‐MEM I Reduced‐Serum Medium (Gibco, 31,985,062) according to the manufacturer's instructions. Cell transfection validation is reported in Figure S1h–k.

### 2.7 Cell Viability and Cell Death Assays

The MTT (3‐[4,5‐dimethylthiazol‐2‐yl]‐2,5 diphenyl tetrazolium bromide) assay was performed to assess cell viability as previously reported (Li et al. 2019; Wong et al. 2020). The MTT assay is based on the ability of mitochondria of living cells to convert MTT (yellow) to formazan (purple). Cells were seeded on a collagen‐coated 96‐well plate (Sigma‐Aldrich, C7661‐10MG). After the specified treatments (as described in section 2.6), 10 μL of MTT (5 mg/mL) was added into each well. Cells were incubated for 3 h in a humidified incubator at 37°C with 5% CO_2_. Media were completely removed and 100 μL of DMSO was added to each well. The plate was read at 490 nm in a microplate reader. Cell viability was expressed as a percentage of the control group.

The propidium iodide (PI) assay was performed to assess cell death as reported before (Chen et al. 2015; Li et al. 2019). PI is a membrane‐impermeant dye that intercalates with nucleic acids, resulting in an increase in fluorescence. Since PI cannot penetrate intact cell membranes, it selectively labels dead cells. Cells were seeded on a collagen‐coated 96‐well plate (Sigma‐Aldrich, C7661‐10MG). After the specified treatments (as described in section 2.6), 1 μL of PI (1 mg/mL) was added to each well. Cells were incubated for 3 h in a humidified incubator at 37°C with 5% CO_2_. To establish a positive control, three wells were treated with 0.2% Triton X‐100 for 15 min to induce 100% cell death. The plate was read at 488/630 nm in a microplate reader. Cell viability was expressed as a percentage of the 100% cell death group.

### 2.8 Phagocytosis Assay

siRNA‐transfected microglial cells were seeded on collagen‐coated 12‐well plates at a density of 2.5 × 10^4^ cells per well and allowed to adhere overnight. In parallel, neurons were differentiated and treated with 100 μM of 6‐OHDA for 24 h to induce neuronal death. Supernatant was collected and washed with PBS. Microglia were added to the neuronal supernatant at a 1:1 ratio for 1.5 h to allow for phagocytosis. Following this, the media were replaced to remove any neurons that were not phagocytized. To assess the degradation of the phagocytized neurons, microglia were incubated for an additional 3 h. The cell media were then removed, and cells were fixed with 4% PFA for 20 min. Cells were permeabilized with 0.1% Triton X‐100 in PB for 20 min. A primary antibody, anti‐beta III tubulin (βIIIT, 1:1000, ab18207), was incubated overnight at 4°C. Cells were washed three times, and the secondary antibody was incubated for 2 h. Cells were incubated with Phalloidin (Alexa Fluor 488 Phalloidin, A1237), a marker for F‐actin, and then DAPI, a blue‐fluorescent DNA stain. Coverslips were mounted on slides with ProLong Gold Antifade Mountant (Invitrogen, P36930). Images were captured in a confocal laser scanning microscope (LSM700 Laser Scanning Microscope, Zeiss, Germany). Experiments were performed in triplicate or quadruplicate, with at least 15 image frames per well captured using a 20× objective. The percentage of phagocytic microglia was calculated by dividing the number of microglia that had engulfed red‐labeled neuronal fragments by the total number of microglia. The number of phagocytic pouches per microglia was counted, and microglia were classified as microglia having one, two, three, four, or five or more pouches.

### 2.9 Quantitative Reverse‐Transcription Polymerase Chain Reaction (RT‐qPCR)

Cells were collected and lysed in 0.6 mL of Lysis buffer (#46–6001, Invitrogen). RNA isolation was performed using the PureLink RNA Mini Kit (#12183018A, Invitrogen). RNA was quantified with the NanoDrop2000 (Thermo Fisher Scientific). Complementary DNA (cDNA) was synthesized with the Superscript IV Reverse Transcriptase (Invitrogen, 18,090,010) using 1000 ng of RNA. Quantitative reverse‐transcription polymerase chain reaction (RT‐qPCR) was performed in a 10 μL volume of total reaction mixture containing 12.5 ng cDNA, 200 nM of each primer, and the PowerTrack SYBR Green Master Mix (#A46109, Applied Biosystems). Primers were designed using the Primer Blast program (https://blast.ncbi.nlm.nih.gov). The following primers were used: TRPM2 (Forward Primer GGCTACATGGATGACCCGAG, Reverse Primer GGCGTGCAGGTTAGAGTTCA), IL‐1β (Forward Primer CAGAAGTACCTGAGCTCGCC, Reverse Primer CCTGGAAGGAGCACTTCATCT), IL‐6 (Forward Primer AGTGAGGAACAAGCCAGAGC; Reverse Primer ATTGCATCTAGATTCTTTGCCTTT), iNOS (Forward Primer TCCCGAGTCAGAGTCACCAT; Reverse Primer CATGCAGACAACCTTGGGGT); GAPDH (Forward Primer CTGGGCTACACTGAGCACC, Reverse Primer AAGTGGTCGTTGAGGGCAATG), TBP (Forward Primer GAGCCAAGAGTGAAGAACAGTC, Reverse Primer GCTCCCCACCATATTCTGAATCT). Plate was run on the QuantStudio 5 Real‐Time PCR System (Applied Biosystems) (2 min at 95°C, 5 s at 95°C for 40 cycles, and 30 s at 60°C). The relative mRNA expression was determined using the ΔΔCt method and normalized to the stable reference genes GAPDH and TBP.

### 2.10 Statistical Analysis

The statistical analysis was performed by using the software Statistical Package for the Social Sciences (SPSS), version 23 (IBM SPSS Statistic, Chicago, IL). Shapiro–Wilk test and Levene's test were used to analyze normality and equality of variance. Animal data (TH% cells, Iba1 and CD68 density, morphology, ELISA, and CD68 area in microglia) were analyzed using the three‐way analysis of variance (ANOVA) test followed by the Bonferroni post hoc test. Three dependent variables were considered: the PD model, the genotype, and the sex. Statistical power calculation was performed for the animal data using SPSS, and the data can be found in Table S4. Cell data were analyzed using the one‐way ANOVA (for validation experiments) or two‐way ANOVA (for MTT, PI, phagocytosis assay, and RT‐qPCR) followed by the Bonferroni post hoc test. The variables considered were: the PD model and knockdown. Statistical significance was accepted at *p* ≤ 0.05. Graphs were designed in GraphPad Prism software 6 (GraphPad Prism, RRID:SCR_002798). Data were presented as violin plot, box plots with min/max whiskers, or bar graphs with means and standard errors of the mean (SEM).

The 19 morphometrical parameters extracted from the Fractal analysis were subjected to a cluster analysis using the two‐steps cluster analysis in SPSS. To do that, data were normalized to obtain similar scale values. Principal component analysis (PCA) was also performed in SPSS to reduce dimensionality. The adequacy of the sample was assessed through the Bartlett sphericity and Kaiser–Meyer–Olkin (KMO) tests. Only components with eigenvalues above one were considered. The components needed to explain over 70% of the accumulative variance. The elbow method was also used to confirm the correct number of extracted components. Each cell values were plotted on a scatter plot designed in SPSS. The cell color code was based on the cluster classification (1 or 2) and on the experimental group (TRPM2+/+; TRPM2+/+ PD; TRPM2+/−; TRPM2+/− PD; TRPM2−/−; TRPM2−/− PD) for male and female animals.

## 3 Results

### 3.1 TRPM2 Deletion Prevents Dopaminergic Neuronal Loss, Microglial and Lysosome‐Associated Protein Density Increase in Male and Female PD‐Induced Mice

**FIGURE 1:**
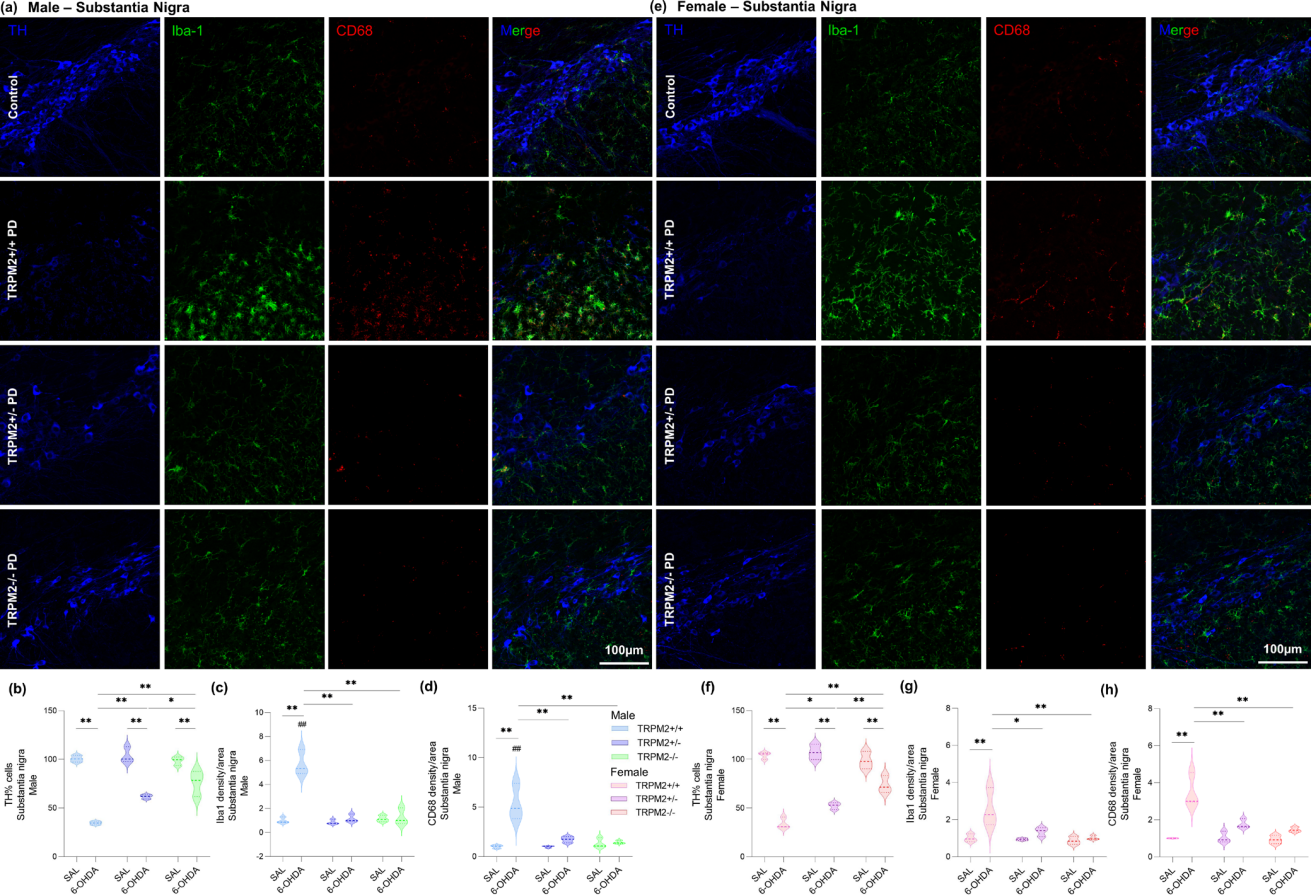
TRPM2 knockout reduced dopaminergic neuronal death, microglia and CD68 density in the substantia nigra pars compacta of the 6‐hydroxydopamine‐injected animals. Representative images from the immunofluorescence assays on males (a) and females (e). The first column illustrates the blue channel (tyrosine hydroxylase—TH) staining. The second column illustrates the green channel (Iba‐1, microglial marker) staining. The third column illustrates the red channel (CD68, lysosome‐associated protein marker). TRPM2+/+ PD: Wild‐type animals that received 6‐OHDA injection; TRPM2+/− PD: Heterozygote animals that received 6‐OHDA injection; and TRPM2−/− PD: Homozygote animals that received 6‐OHDA injection. As no difference was found comparing the three vehicle genotypes (TRPM2+/+, TRPM2+/−, and TRPM2−/−), we choose to illustrate just one group that is pointed as control in the representative image. Percentage of tyrosine hydroxylase (TH)‐positive cells in substantia nigra in males (b) and females (f). Integrated density of Iba‐1 in males (c) and females (g). Integrated density of CD68 in males (d) and females (h). The graphs show violin plots. *N* = 3 per group. *Comparison between groups of the same sex; #comparison of males and females. **p < 0.01; *p < 0.05; ^##^ *p* < 0.01.

Here we designed an experimental paradigm aimed to explore the potential effect of TRPM2 absence in PD‐induced animal and cell models, with a particular focus on the well‐known contribution of microglia to PD pathology. To this end, we first injected male and female wild‐type, TRPM2‐null, and TRPM2‐heterozygote mice with 6‐OHDA to induce the PD model. As we showed before (Ferreira, Ulrich, Feng, et al. 2024a), the absence of TRPM2 alleviated dopaminergic neuronal death. In Figure 1a,b,e,f, we show the staining of tyrosine hydroxylase (TH), a key enzyme in dopamine synthesis, in the SNc of male and female animals. The PD model induction was confirmed here, since a reduction to around 30% in the number of TH+ cells was noted in the TRPM2+/+ PD group when compared to the TRPM2+/+ group from both male and female mice {*Injection × Genotype* [F(2,24) = 31.274; *p* = 0.000]; *p* = 0.000 for both comparisons}. In addition, *Trpm2* partial and complete genetic deletion alleviated dopaminergic neuronal loss in both male and female (*p* < 0.01 for all comparisons). No difference was observed when comparing male and female mice (Male: TRPM2+/+: 100.00 ± 1.99, TRPM2+/+ PD: 34.34 ± 1.60; TRPM2+/−: 103.12 ± 4.68; TRPM2+/− PD: 61.20 ± 1.29; TRPM2 −/−: 97.97 ± 2.57; TRPM2 −/− PD: 75.41 ± 7.56; Female: TRPM2+/+: 100.00 ± 2.04; TRPM2+/+ PD: 32.67 ± 3.24; TRPM2+/−: 103.20 ± 4.37; TRPM2+/− PD: 50.28 ± 1.99; TRPM2−/−: 95.01 ± 4.96; TRPM2−/− PD: 70.82 ± 4.94).

**FIGURE 2:**
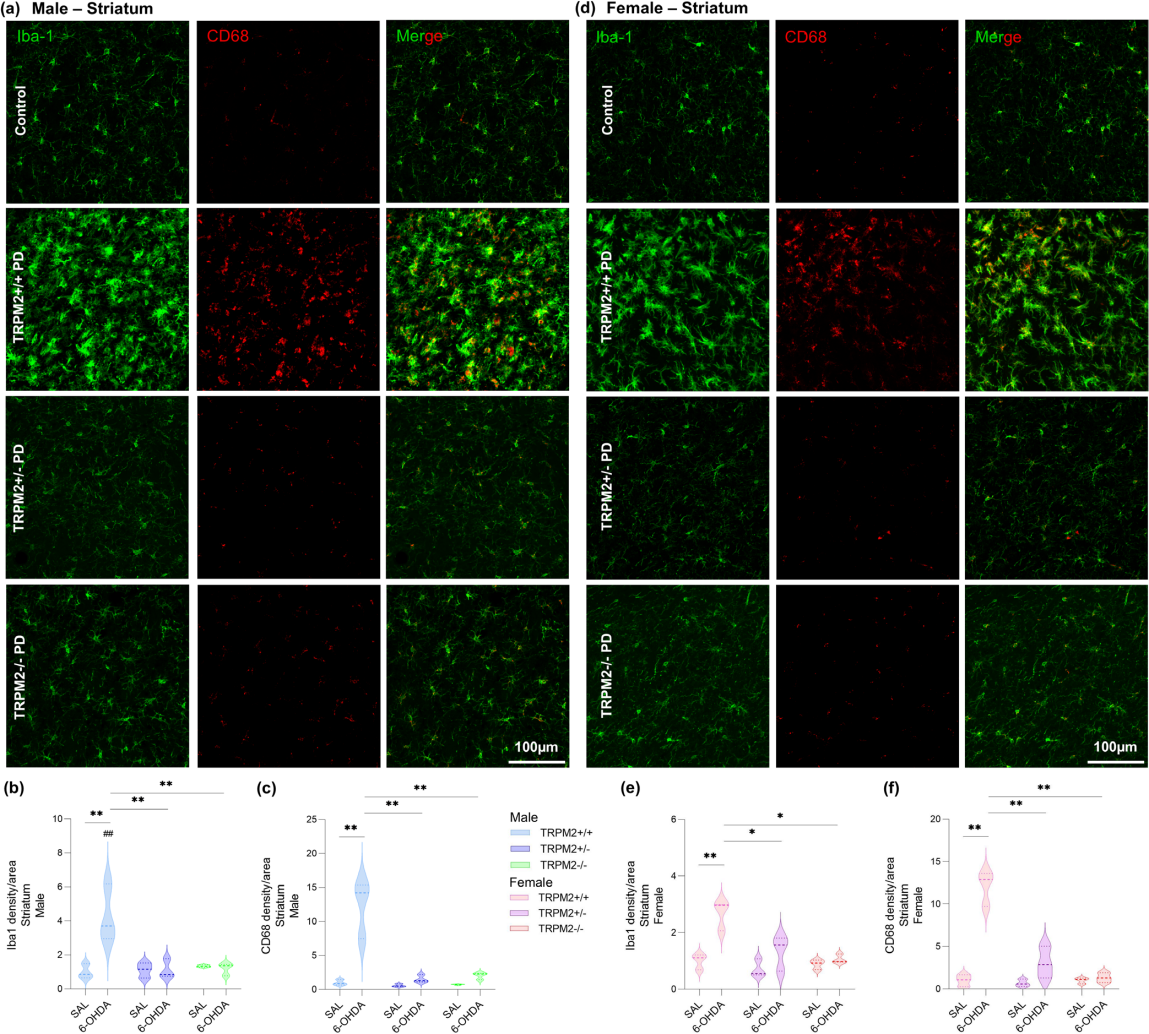
TRPM2 knockout reduced microglia and CD68 density in the striatum of the 6‐hydroxydopamine‐injected animals. Representative images from the immunofluorescence assays on males (a) and females (d). The first column illustrates the green channel (Iba‐1, microglial marker) staining. The second column illustrates the red channel (CD68, lysosome‐associated protein marker). TRPM2+/+ PD: Wild‐type animals that received 6‐OHDA injection; TRPM2+/− PD: Heterozygote animals that received 6‐OHDA injection; and TRPM2−/− PD: Homozygote animals that received 6‐OHDA injection. As no difference was found comparing the three vehicle genotypes (TRPM2+/+, TRPM2+/−, and TRPM2−/−), we choose to illustrate just one group that is pointed as control in the representative image. Integrated density of Iba‐1 in males (b) and females (e). Integrated density of CD68 in males (c) and females (f). The graphs show violin plots. *N* = 3 per group. *Comparison between groups of the same sex; #comparison of males and females. **p < 0.01; *p < 0.05; ^##^ *p* < 0.01.

After the model validation, we proceeded to analyze the effects of TRPM2 genetic deletion on microglia. First, Iba1 density was assessed in both the SNc (Figure 1c,g) and the CPu (Figure 2b,e). It was noted an increase in the TRPM2+/+ PD compared to the TRPM2+/+ group from both male and female mice in the SNc {*Interaction* [F(11,24) = 9.887; *p* = 0.001]; *p* = 0.000 for male and *p* = 0.001 for female} and in the CPu {*Injection × Genotype* [F(2,24) = 14.119; *p* = 0.000]; *p* = 0.000 for male and *p* = 0.003 for female}. This indicates enhanced microglia density in the wild‐type PD‐induced group, as expected. A difference between male and female was observed, with an increase in Iba1 staining in TRPM2+/+ PD male compared to TRPM2+/+ PD female (*p* = 0.000 for the SNc and *p* = 0.004 for the CPu). The analysis also revealed a decrease in Iba1 staining in the TRPM2+/− PD and TRPM2−/− PD groups, in both the SNc and the CPu from male and female, with no differences in comparison to their respective controls (SNc: Male: TRPM2+/+: 1.00 ± 0.15, TRPM2+/+ PD: 5.72 ± 0.61; TRPM2+/−: 0.86 ± 0.12; TRPM2+/− PD: 1.15 ± 0.19; TRPM2 −/−: 1.14 ± 0.16; TRPM2 −/− PD: 1.26 ± 0.40; Female: TRPM2+/+: 1.00 ± 0.12; TRPM2+/+ PD: 2.58 ± 0.60; TRPM2+/−: 0.94 ± 0.04; TRPM2+/− PD: 1.35 ± 0.14; TRPM2−/−: 0.87 ± 0.13; TRPM2−/− PD: 1.02 ± 0.07; CPu: Male: TRPM2+/+: 1.00 ± 0.25, TRPM2+/+ PD: 4.27 ± 0.97; TRPM2+/−: 1.10 ± 0.26; TRPM2+/− PD: 1.34 ± 0.05; TRPM2 −/−: 1.20 ± 0.21; TRPM2 −/− PD: 1.26 ± 0.40; Female: TRPM2+/+: 1.00 ± 0.16; TRPM2+/+ PD: 2.67 ± 0.30; TRPM2+/−: 0.71 ± 0.18; TRPM2+/− PD: 1.34 ± 0.36; TRPM2−/−: 0.88 ± 0.10; TRPM2−/− PD: 1.06 ± 0.09).

We also analyzed CD68 staining in both the SNc (Figure 1d,h) and the CPu (Figure 2c,f). CD68 is a lysosome‐associated protein frequently used as a marker of phagocytosis (Chistiakov et al. 2017; Hwang et al. 2022), and thus its increase suggests enhanced microglial phagocytic activity. An increase in CD68 staining was detected in TRPM2+/+ PD in comparison to the TRPM2+/+ group from both male and female mice in the SNc {*Injection × Genotype* [F(2,24) = 21.448; *p* = 0.000]; *p* = 0.000 for both comparisons} and in the CPu {*Injection × Genotype* [F(2,24) = 43.017; *p* = 0.000]; *p* = 0.000 for both comparisons}. This suggests an increase in microglial activation after the PD model induction. We also observed an increase in CD68 staining in wild‐type PD‐induced male in comparison to female mice, but only in the SNc (*p* = 0.001). Additionally, the increase of CD68 density was not observed in the TRPM2+/− PD and TRPM2−/− PD groups, from both sex and brain regions (SNc: Male: TRPM2+/+: 1.00 ± 0.10, TRPM2+/+ PD: 5.37 ± 1.06; TRPM2+/−: 0.98 ± 0.12; TRPM2+/− PD: 1.71 ± 0.18; TRPM2−/−: 1.33 ± 0.29; TRPM2 −/− PD: 1.44 ± 0.10; Female: TRPM2+/+: 1.00 ± 0.01; TRPM2+/+ PD: 3.51 ± 0.52; TRPM2+/−: 1.04 ± 0.18; TRPM2+/− PD: 1.77 ± 0.15; TRPM2−/−: 0.93 ± 0.13; TRPM2 −/− PD: 1.47 ± 0.08; CPu: Male: TRPM2+/+: 1.00 ± 0.24, TRPM2+/+ PD: 12.34 ± 2.46; TRPM2+/−: 0.59 ± 0.13; TRPM2+/− PD: 1.53 ± 0.34; TRPM2−/−: 0.73 ± 0.05; TRPM2−/− PD: 2.03 ± 0.30; Female: TRPM2+/+: 1.00 ± 0.41; TRPM2+/+ PD: 12.07 ± 1.19; TRPM2+/−: 0.68 ± 0.25; TRPM2+/− PD: 3.07 ± 1.08; TRPM2−/−: 0.99 ± 0.20; TRPM2−/− PD: 1.32 ± 0.34). It is important to note that Iba1 and CD68 staining extended beyond TH‐positive regions. Previous studies have shown that microglial activation precedes dopaminergic neuron loss, begins in specific areas of the SNc, and persists throughout the degenerative process (Marinova‐Mutafchieva et al. 2009; Virgone‐Carlotta et al. 2013). Considering that this is a progressive model and that TH‐positive neuronal loss is already evident at the time point analyzed, it is likely that the inflammatory response has extended into nondopaminergic regions as well.

### 3.2 Microglial Morphology Is Maintained in TRPM2‐Knockout Male and Female Mice After PD Induction

**FIGURE 3:**
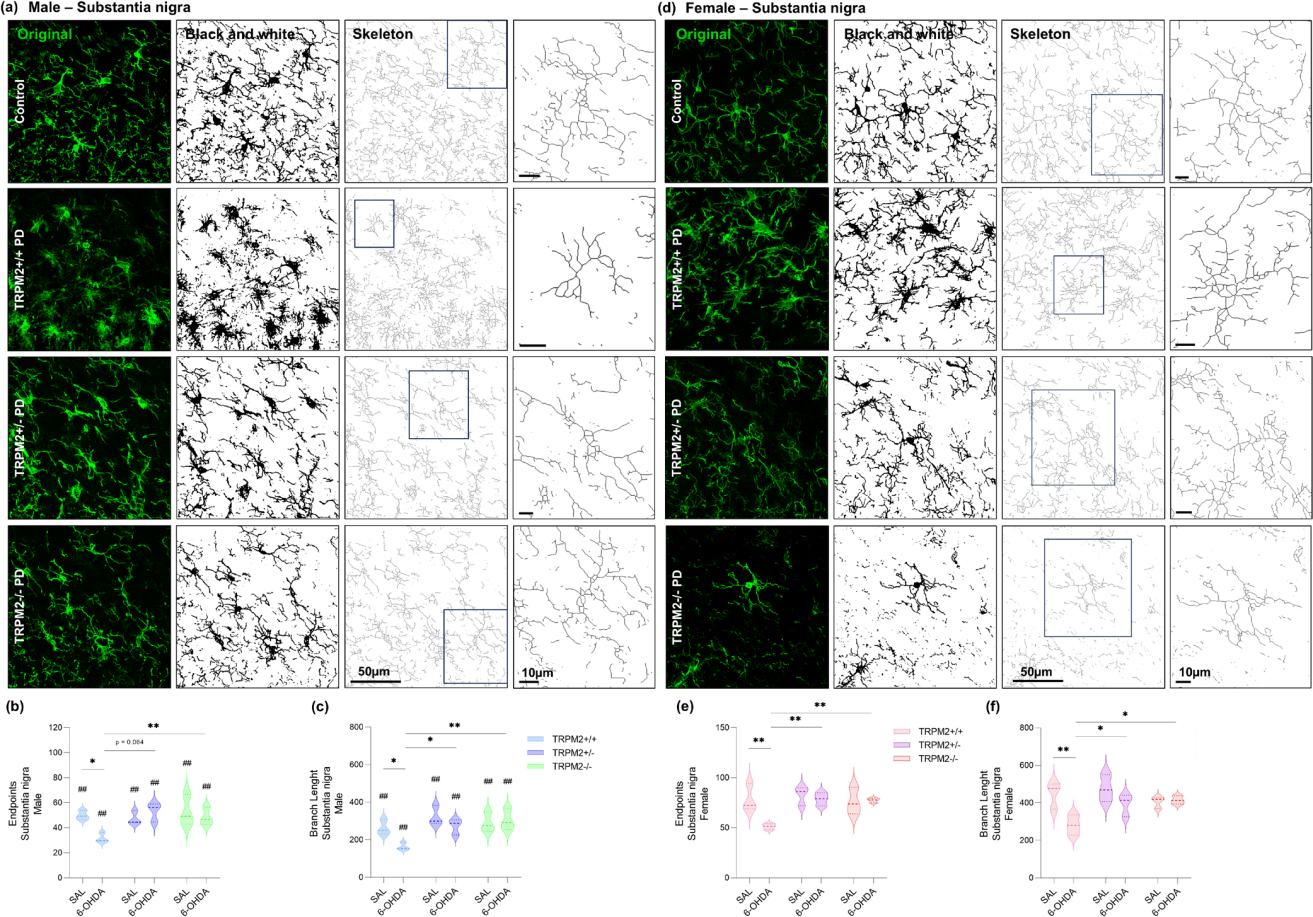
Skeleton analysis of microglial cells in the substantia nigra pars compacta. Representative images from the immunofluorescence assays and Skeleton analysis on males (a) and females (d). The first column illustrates the original green channel images of Iba‐1 staining. The second column illustrates the black and white binary image. The third column illustrates the skeletonized image and the fourth column illustrates a zoomed cell from the Skeleton analysis. TRPM2+/+ PD: Wild‐type animals that received 6‐OHDA injection; TRPM2+/− PD: Heterozygote animals that received 6‐OHDA injection; and TRPM2−/− PD: Homozygote animals that received 6‐OHDA injection. As no difference was found comparing the three vehicle genotypes (TRPM2+/+, TRPM2+/−, and TRPM2−/−), we choose to illustrate just one group that is pointed as control in the representative image. Graphs of male microglia endpoints/cell (b) and branch length/cell (c). Graphs of female microglia endpoints/cell (e) and branch length/cell (f). The graphs show violin plots. *N* = 3 per group. *Comparison between groups of the same sex; #comparison of males and females. **p < 0.01; *p < 0.05; ^##^ *p* < 0.01.

**FIGURE 4:**
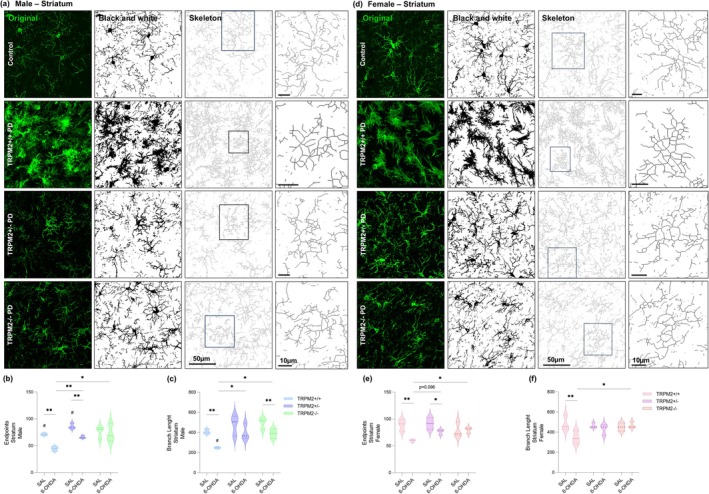
Skeleton analysis of microglial cells in the striatum. Representative images from the immunofluorescence assays and Skeleton analysis on males (a) and females (d). The first column illustrates the original green channel images of Iba‐1 staining. The second column illustrates the black and white binary image. The third column illustrates the skeletonized image and the fourth column illustrates a zoomed cell from the Skeleton analysis. TRPM2+/+ PD: Wild‐type animals that received 6‐OHDA injection; TRPM2+/− PD: Heterozygote animals that received 6‐OHDA injection; and TRPM2−/− PD: Homozygote animals that received 6‐OHDA injection. As no difference was found comparing the three vehicle genotypes (TRPM2+/+, TRPM2+/−, and TRPM2−/−), we choose to illustrate just one group that is pointed as control in the representative image. Graphs of male microglia endpoints/cell (b) and branch length/cell (c). Graphs of female microglia endpoints/cell (e) and branch length/cell (f). The graphs show violin plots. *N* = 3 per group. *Comparison between groups of the same sex; #comparison of males and females. **p < 0.01; *p < 0.05; ^##^ *p* < 0.01.

Another relevant aspect of microglia is its morphology, which is highly diverse and has been shown to be altered in neurodegenerative diseases (Arnanz et al. 2025; Vidal‐Itriago et al. 2022). We first assessed the number of endpoints and branch length in the SNc (Figure 3) and in the CPu (Figure 4), as a measure of overall microglia ramification through the Skeleton analysis. As observed in our previous study (Ferreira, Ulrich, Feng, et al. 2024a), a decrease in the number of endpoints (Figure 3b,e, Figure 4b,e) and in the branch length (Figure 3c,f, Figure 4c,f) was found in the TRPM2+/+ PD groups, in both male and female, in the SNc {Endpoints: *Injection × Genotype* [F(2,24) = 7.883; *p* = 0.002]; *p* = 0.014 for males and *p* = 0.001 for females; Branch length: *Injection × Genotype* [F(2,24) = 6.083; *p* = 0.007]; *p* = 0.024 for males and *p* = 0.001 for females} and the CPu {Endpoints: *Injection × Genotype* [F(2,24) = 4.394; *p* = 0.024]; *p* = 0.001 for males and *p* = 0.000 for females; Branch length: *Injection* [F(1,24) = 18.498; *p* = 0.000]; *Genotype* [F(2,24) = 7.040; *p* = 0.004]; *p* = 0.003 for males and *p* = 0.008 for females}. This indicates shorter and fewer ramifications in the wild‐type PD animals compared to the control group. In addition, when we compared the number of endpoints and branch length in the SNc, all female experimental groups showed longer and higher ramifications than males (Endpoints: *p* < 0.01 for all comparison; branch length; *p* < 0.02 for all comparisons). However, in the CPu, for the number of endpoints only the TRPM2+/+ (*p* = 0.021) and TRPM2+/− (*p* = 0.037) groups and for the branch length only the TRPM2+/+ PD (*p* = 0.049) group showed an increase when comparing females to males. *Trpm2* partial and complete deletion in 6‐OHDA‐injected male and female mice exhibited values similar to the control groups and higher than the wild‐type PD group (Mean and SEM values for these parameters can be found in Table S1).

**FIGURE 5:**
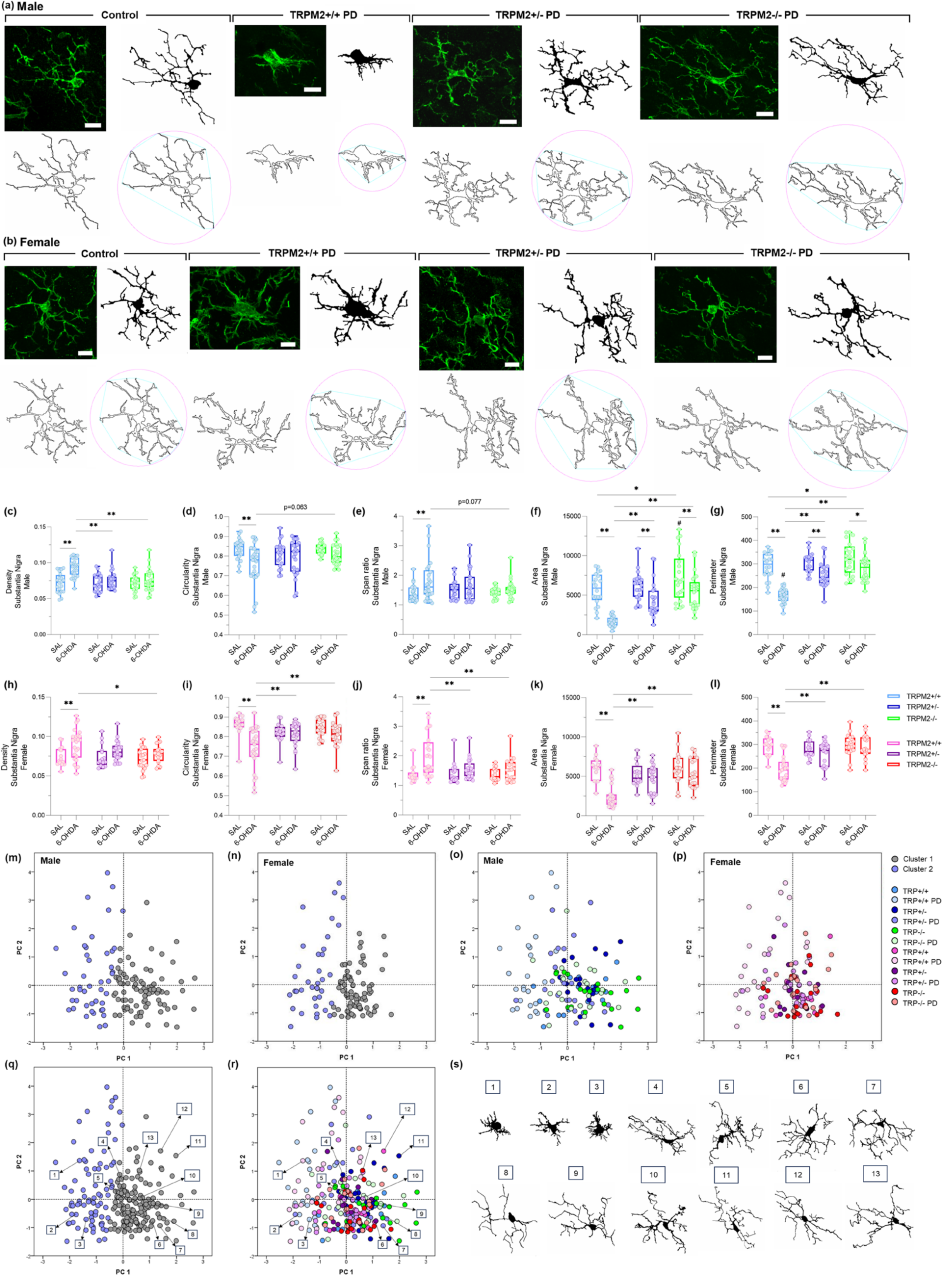
Fractal analysis, cluster analysis, and principal components analysis (PCA) of microglial cells in the substantia nigra pars compacta. Representative images from the Fractal analysis of males (a) and females (b). Representative images illustrate the original cell (top left), the filled (top right), the outline (bottom left), and the convex hull with the bounding circle (bottom right). TRPM2+/+ PD: Wild‐type animals that received 6‐OHDA injection; TRPM2+/− PD: Heterozygote animals that received 6‐OHDA injection; and TRPM2−/− PD: Homozygote animals that received 6‐OHDA injection. As no difference was found comparing the three vehicle genotypes (TRPM2+/+, TRPM2+/−, and TRPM2−/−), we choose to illustrate just one group that is pointed as control in the representative image. Graphs of morphometric parameters extracted for males and females, respectively: Density (c) and (h), circularity (d) and (i), span ratio (e) and (j), area (f) and (k), perimeter (g) and (l). The graphs show box plots with min/max whiskers and each dot represents the data from each analyzed cell. *N* = 3 animals per group. The exact number of cells analyzed can be found in the Materials and Methods section. *Comparison between groups of the same sex; #comparison of males and females. **p < 0.01; *p < 0.05; ^#^ *p* < 0.05. Scatter plots showing the distribution of male (m and o), female (n and p), and both sexes (q and r) microglia cells in the principal component plane. Each cell was color coded according to the cluster allocation (m, n, and q) or to the experimental group to which they belong (o, p, and r). Different microglia‐filled shapes from each experimental group are illustrated on (s).

**FIGURE 6:**
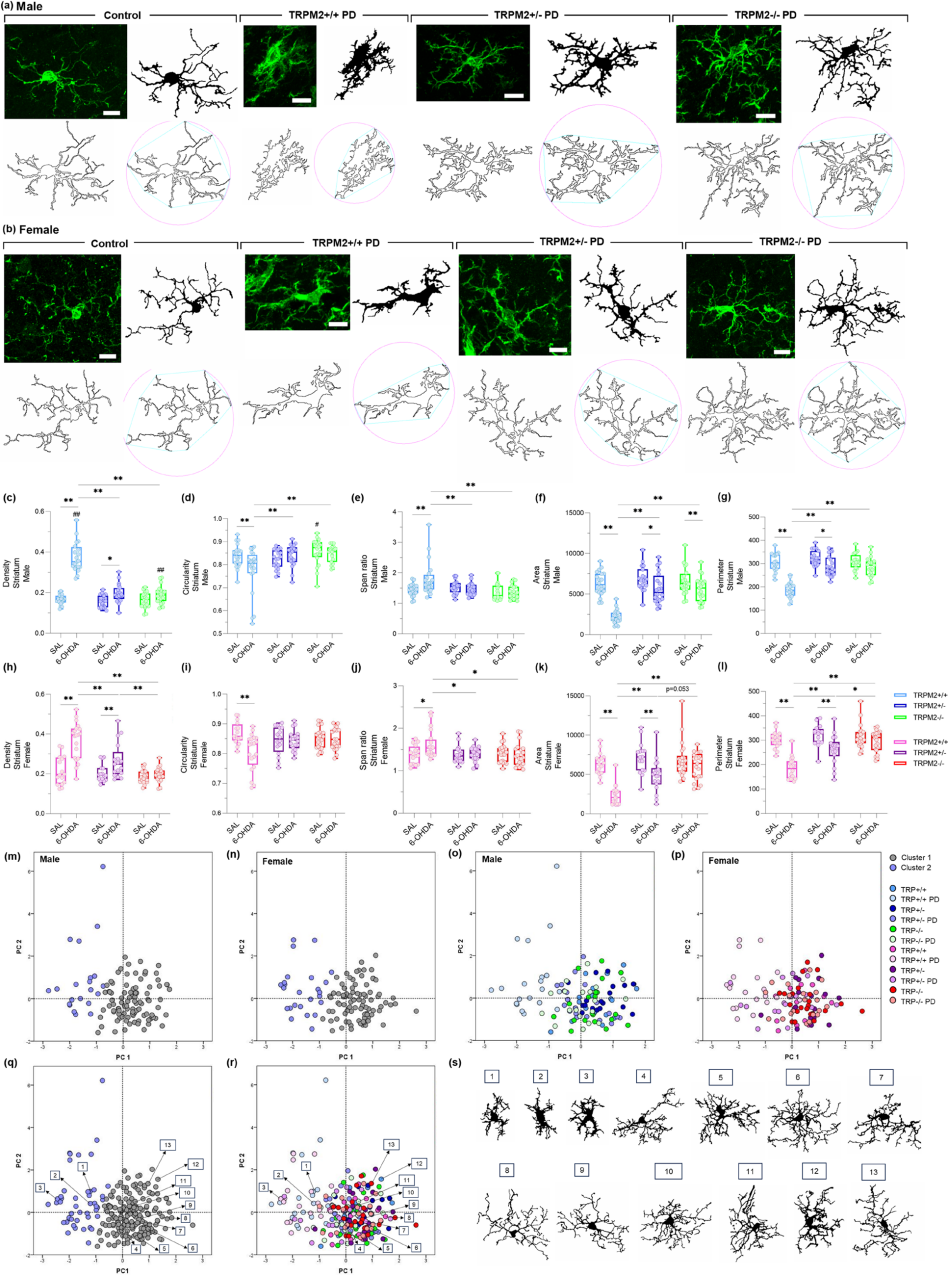
Fractal analysis, cluster analysis, and principal components analysis (PCA) of microglial cells in the striatum. Representative images from the Fractal analysis of males (a) and females (b). Representative images illustrate the original cell (top left), the filled (top right), the outline (bottom left), and the convex hull with the bounding circle (bottom right). TRPM2+/+ PD: Wild‐type animals that received 6‐OHDA injection; TRPM2+/− PD: Heterozygote animals that received 6‐OHDA injection; and TRPM2−/− PD: Homozygote animals that received 6‐OHDA injection. As no difference was found comparing the three vehicle genotypes (TRPM2+/+, TRPM2+/−, and TRPM2−/−), we chose to illustrate just one group that is pointed out as control in the representative image. Graphs of morphometric parameters extracted for males and females, respectively: Density (c) and (h), circularity (d) and (i), span ratio (e) and (j), area (f) and (k), perimeter (g) and (l). The graphs show box plots with min/max whiskers, and each dot represents the data from each analyzed cell. *N* = 3 animals per group. The exact number of cells analyzed can be found in the Materials and Methods section. *Comparison between groups of the same sex; #comparison of males and females. **p < 0.01; *p < 0.05; ^#^ *p* < 0.05. Scatter plots showing the distribution of male (m and o), female (n and p), and both sexes (q and r) microglia cells in the principal component plane. Each cell was color‐coded according to the cluster allocation (m, n, and q) or to the experimental group to which they belong (o, p, and r). Different microglia‐filled shapes from each experimental group are illustrated on (s).

We then analyzed individual cells with the Fractal analysis as a more specific method to identify morphometric differences between cells. Nineteen parameters were extracted. For simplicity, a selection of five of these parameters is shown in Figure 5, for the SNc, and in Figure 6, for the CPu, but all of them are available in Table S2. All of the parameters, Density (Figure 5c,h, Figure 6c,h), Circularity (Figure 5d,i, Figure 6d,i), Span ratio (Figure 5e,j, Figure 6e,j), Area (Figure 5f,k, Figure 6f,k), and Perimeter (Figure 5g,l, Figure 6g,i), showed alterations in the wild‐type 6‐OHDA‐injected animals when compared to the wild‐type control group {SNc: Density: *Injection × Genotype* [F(2,239) = 6.131; *p* = 0.003]; *p* = 0.000 for males and *p* = 0.001 for females; Circularity: *Injection × Genotype* [F(2,239) = 8.011; *p* = 0.000]; p = 0.000 for both sex; Span ratio: *Injection × Genotype* [F(2,239) = 5.102; *p* = 0.007]; p = 0.003 for males and p = 0.000 for females; Area: *Injection × Genotype* [F(2,239) = 12.734; *p* = 0.000]; *Injection × Sex* [F(2,239) = 5.054; *p* = 0.025], *p* = 0.000 for both sex; Perimeter: *Injection × Genotype* [F(2,239) = 19.285; *p* = 0.000]; *Injection × Sex* [F(2,239) = 5.870; *p* = 0.016]; *p* = 0.000 for both sex; CPu: Density: *Interaction* [F(11,219) = 5.812; *p* = 0.003]; *p* = 0.000 for both sex; Circularity: *Injection × Genotype* [F(2,219) = 9.467; *p* = 0.000]; *p* = 0.000 for males and *p* = 0.001 for females; Span ratio: *Injection × Genotype* [F(2,219) = 9.546; *p* = 0.000]; *p* = 0.000 for males and *p* = 0.012 for females; Area: *Injection × Genotype* [F(2,219) = 18.884; *p* = 0.000]; p = 0.000 for both sex; Perimeter: *Injection × Genotype* [F(2,219) = 28.476; *p* = 0.000]; *p* = 0.000 for both sex}. As expected, these results indicate that microglia from the PD group are more dense (higher cell compaction), elongated, have reduced circularity, and have lower area and perimeter (indicative of fewer ramifications). These alterations were not observed to the same extent in the TRPM2 knockout animals. In the SNc of males, density, circularity, and span ratio were not different from the respective controls. In the SNc of females, density, circularity, and span ratio were not different from respective controls. In addition, despite being different from their controls, the area and perimeter of the TRPM2+/− PD (Area: *p* = 0.000; Perimeter: *p* = 0.000) and TRPM2−/− PD (Area: p = 0.000; Perimeter: *p* = 0.000) male group were higher than the TRPM2+/+ PD group, as well as for almost all of the parameters assessed. In the CPu of males and females, circularity and span ratio from TRPM2 partial and complete deletion were not different from respective controls. In addition, despite some differences when compared to their controls, microglia from the TRPM2+/− PD and TRPM2−/− PD groups exhibited lower density (male: *p* = 0.000 for both genotypes; female: *p* = 0.000 for both genotypes) and span ratio (male: *p* = 0.000 for both genotypes; female: *p* = 0.020 for TRPM2+/− PD and *p* = 0.006 for TRPM2−/− PD), and higher circularity (male: *p* = 0.000 for both genotypes), area (male: *p* = 0.000 for both genotypes; female: *p* = 0.000 for both genotypes), and perimeter (male: *p* = 0.000 for both genotypes; female: *p* = 0.000 for both genotypes), in the majority of the comparisons to the TRPM2+/+ PD group. Overall, no differences were observed when comparing male and female groups. It seems that TRPM2 absence restores microglial morphology to that found in control mice, which corresponds to a homeostatic‐like phenotype, in both male and female animals.

### 3.3 Microglia Are Divided Into Two Different Cell Clusters

Given the microglia alterations revealed by the Fractal analysis, a cluster analysis was performed to identify possible clusters of cells in the SNc (Figure 5) and in the CPu (Figure 6). It was observed that microglia can be split into two clusters. In the SNc, 82.9% of the cells were allocated in cluster 1 and 17.1% in cluster 2 for the TRPM2+/+ group; 97.2% of the cells were allocated in cluster 1 and 2.8% in cluster 2 for the TRPM2+/− group; 87.5% of the cells were allocated in cluster 1 and 12.5% in cluster 2 for the TRPM2−/− group; 10% of the cells were allocated in cluster 1 and 90% in cluster 2 for the TRPM2+/+ PD group; 65.2% of the cells were allocated in cluster 1 and 34.8% in cluster 2 for the TRPM2+/− PD group; 81.8% of the cells were allocated in cluster 1 and 18.2% in cluster 2 for the TRPM2−/− PD group. In the CPu, 100% of the cells were allocated in cluster 1 for the TRPM2+/+ group; 97.1% of the cells were allocated in cluster 1 and 2.9% in cluster 2 for the TRPM2+/− group; 100% of the cells were allocated in cluster 1 for the TRPM2−/− group; 10% of the cells were allocated in cluster 1 and 90% in cluster 2 for the TRPM2+/+ PD group; 86.8% of the cells were allocated in cluster 1 and 13.2% in cluster 2 for the TRPM2+/− PD group; 90% of the cells were allocated in cluster 1 and 10% in cluster 2 for the TRPM2−/− PD group.

Next, a PCA analysis was carried out with all 19 parameters to reduce dimensionality. Two components were identified for both regions. For the SNc, principal component (PC) 1 accounted for 50.63% of the observed variability and PC2 accounted for 24.67%. For the CPu, PC1 accounted for 51.22% of the observed variability and PC2 accounted for 21.66%. Scatter plot diagrams were built using the PC scores of the cells and the cluster allocation (for the SNc, Figure 5m,n,q; for the CPu, Figure 6m,n,q) or the experimental group allocation (for the SNc, Figure 5o,p,r; for the CPu, Figure 6o,p,r). Some representative microglia from each experimental group can be observed in Figure 5s, for the SNc, and in Figure 6s, for the CPu. Microglia in cluster 1 exhibited reduced branch length and number of endpoints, smaller cell area and perimeter, lower circularity, and increased cell density. These morphological features are characteristic of a reactive phenotype, often associated with enhanced phagocytic activity and pro‐inflammatory responses. In contrast, microglia in cluster 2 displayed more complex and extended processes, lower cell density, larger area and perimeter, and increased circularity, consistent with a homeostatic or surveillant phenotype. Notably, microglia from the TRPM2+/− PD and TRPM2−/− PD groups showed morphological profiles closely resembling those of the control groups, clustering predominantly within the homeostatic phenotype (cluster 2). In contrast, microglia from the TRPM2+/+ PD group were largely distinct, exhibiting reactive morphology and clustering predominantly within the reactive phenotype (cluster 1).

### 3.4 Increase in Pro‐Inflammatory Cytokines Is Not Observed in the TRPM2‐Knockout Male and Female Mice After PD Induction

**FIGURE 7:**
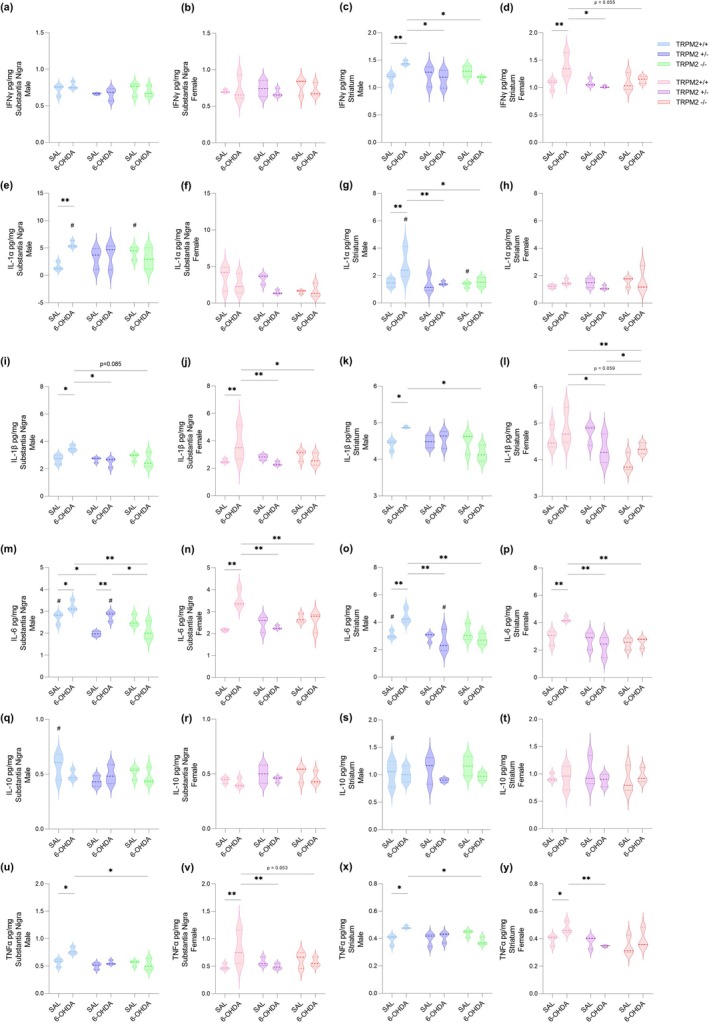
Quantification of cytokines in protein extracts from the midbrain and striatum by multiplex ELISA. Six cytokines were measured in the midbrain of males and females, respectively: IFNγ (a and b), IL‐1α (e and f), IL‐1β (i and j), IL‐6 (m and n), IL‐10 (q and r), and TNFα (u and v). Six cytokines were measured in the striatum of males and females, respectively: IFNγ (c and d), IL‐1α (g and h), IL‐1β (k and l), IL‐6 (o and p), IL‐10 (s and t), and TNFα (x and y). The graphs show a violin plot. *N* = 3 animals per group. *Comparison between groups of the same sex; #comparison of males and females. **p < 0.01; *p < 0.05; ^#^ *p* < 0.05.

In an attempt to get a more complete picture of the inflammation in our model, cytokines were quantified by multiplex ELISA (Figure 7). Six cytokines were assessed: IFNγ, IL‐1α, IL‐1β, IL‐6, IL‐10, and TNFα. In the SNc, no differences were observed in IFNγ {*Injection × Genotype* [F(2,24) = 0.865; *p* = 0.434]}; and IL‐10 levels {*Injection × Genotype* [F(2,24) = 0.682; *p* = 0.515]}. However, IL‐1α, IL‐1β, IL‐6, and TNFα cytokine levels were increased in the TRPM2+/+ 6‐OHDA‐injected male animals and IL‐1β, IL‐6, and TNFα cytokine levels were increased in the TRPM2+/+ 6‐OHDA‐injected female animals {IL‐1α: *Interaction* [F(11,24) = 3.950; *p* = 0.033]; IL‐1β: *Injection × Genotype* [F(2,24) = 7.886; *p* = 0.002]; IL‐6: *Interaction* [F(11,24) = 8.173; *p* = 0.002]; TNFα: *Injection × Genotype* [F(2,24) = 6.332; *p* = 0.006]}. In the CPu, no difference was observed in IL‐10 levels {*Injection × Genotype* [F(2,24) = 0.664; *p* = 0.524]}. On the other hand, the CPu of the male TRPM2+/+ PD showed increased levels of IFNγ, IL‐1α, IL‐1β, IL‐6, and TNFα, while the female TRPM2+/+ PD presented increased levels of IFNγ, IL‐6, and TNFα {IFNγ: *Injection × Genotype* [F(2,24) = 8.923; *p* = 0.001]; IL‐1α: *Injection × Genotype* [F(2,24) = 2.978; *p* = 0.070]; IL‐1β: *Interaction* [F(11,24) = 3.954; *p* = 0.033]; IL‐6: *Injection × Genotype* [F(2,24) = 9.877; *p* = 0.001]; TNFα: *Injection × Genotype* [F(2,24) = 4.835; *p* = 0.017]}. Thus, we confirmed an inflammatory response due to the 6‐OHDA injections in the wild‐type mice of both sexes, and interestingly males presented more differences than females. In addition, almost all cytokine levels were similar (except for IL‐6 TRPM2+/− PD male in the SNc) to respective controls in TRPM2+/− PD and TRPM2−/− PD male and female animals, and lower than the TRPM2+/+ PD group, suggesting that TRPM2 might be involved in the inflammatory response induced by 6‐OHDA (Mean and SEM values for cytokine levels can be found in Table S3).

### 3.5 Area of the Lysosome‐Associated Protein, CD68, Is Reduced in Microglia of TRPM2‐Knockout Male and Female Mice After PD Induction

**FIGURE 8:**
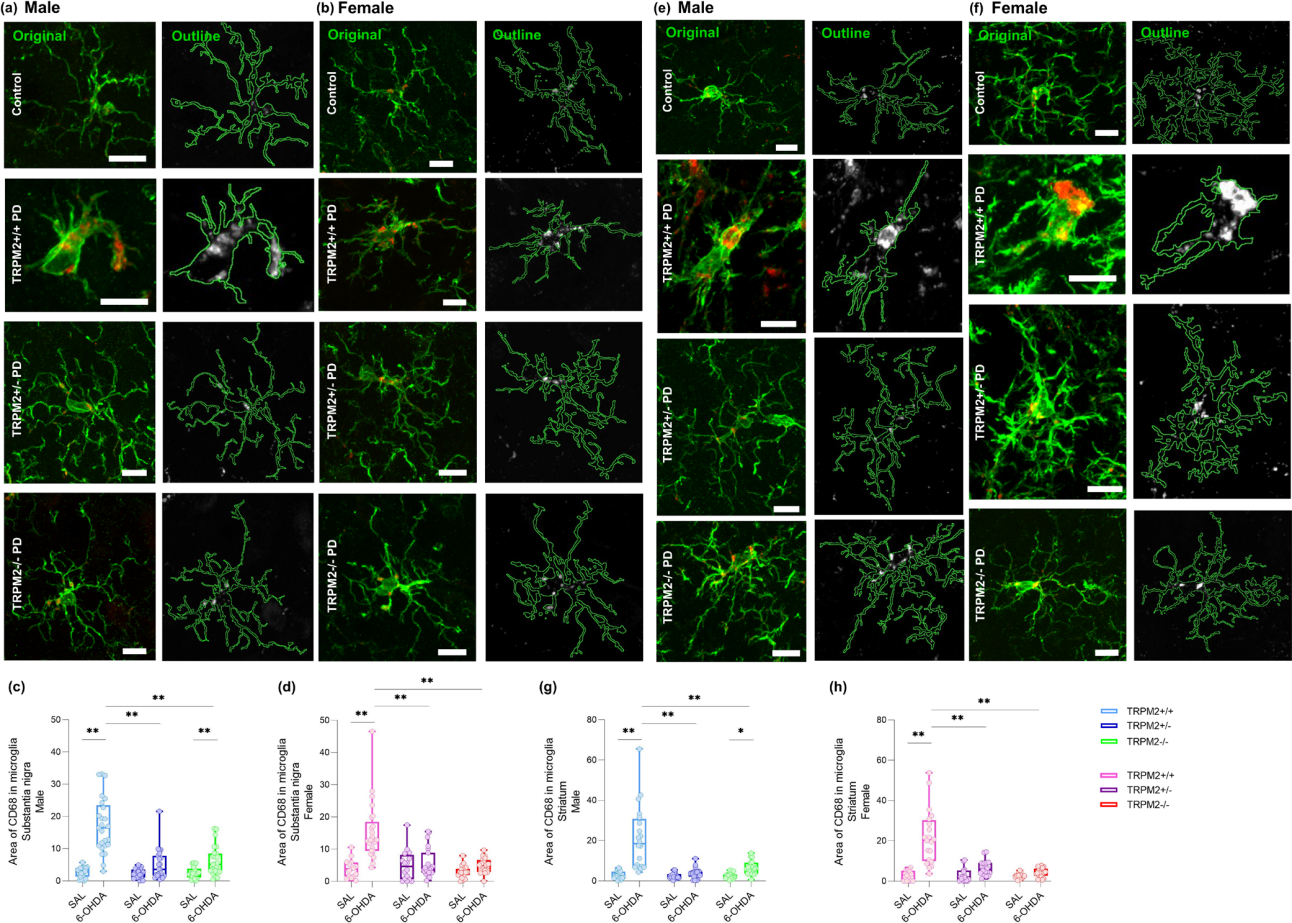
Area of the lysosome‐associated protein, CD68, in substantia nigra and striatum microglia. Representative images from the substantia nigra of males (a) and females (b). Representative images from the striatum of males (e) and females (f). Original images immunolabeled with Iba1 (green) and microglial lysosome marker CD68 (red) are represented in the first column. Microglial outline (green) and CD68 staining (white) in the second column. TRPM2+/+ PD: Wild‐type animals that received 6‐OHDA injection; TRPM2+/− PD: Heterozygote animals that received 6‐OHDA injection; and TRPM2−/− PD: Homozygote animals that received 6‐OHDA injection. As no difference was found comparing the three vehicle genotypes (TRPM2+/+, TRPM2+/−, and TRPM2−/−), we choose to illustrate just one group that is pointed as control in the representative image. Graphs show the quantification of the area of CD68 in Iba‐1‐labeled microglia cells in the substantia nigra of males (c) and females (d) and in the striatum of males (g) and females (h). The graphs show box plots with min/max whiskers and each dot represents the data from each analyzed cell. *N* = 3 animals per group. The exact number of cells analyzed can be visualized in the Materials and Methods section. *Comparison between groups of the same sex; ^#^comparison of males and females. **p < 0.01; *p < 0.05; ^#^ *p* < 0.05.

A second analysis was performed to assess a more functional aspect of each microglial cell. We measured the CD68 area in individual microglial cells (Figure 8). CD68 staining showed a dot‐like granular pattern, consistent with phagocytic vesicles (Figure 8a,b,e,f). In the TRPM2+/+ PD group, the area of CD68 was larger in both the SNc and the CPu of males and females (*p* = 0.000, for all comparisons), suggesting an enhancement in microglial phagocytic activity after PD induction {SNc: *Injection × Genotype* [F(2,239) = 32.387; *p* = 0.000]; CPu: *Injection × Genotype* [F(2,219) = 37.564; *p* = 0.000]}. Additionally, a decrease in microglial CD68 area was observed in TRPM2 partial and complete deletion PD groups when compared to wild‐type PD animals (*p* = 0.000, for all comparisons), suggesting that TRPM2 is involved in microglial phagocytic activity. No differences were noted between males and females (SNc: Male: TRPM2+/+: 2.57 ± 0.37, TRPM2+/+ PD: 17.09 ± 1.71; TRPM2+/−: 2.21 ± 0.32; TRPM2+/− PD: 5.02 ± 1.00; TRPM2−/−: 2.51 ± 0.39; TRPM2−/− PD: 6.20 ± 0.90; Female: TRPM2+/+: 3.92 ± 0.75; TRPM2+/+ PD: 15.13 ± 1.78; TRPM2+/−: 4.96 ± 1.20; TRPM2+/− PD: 5.47 ± 0.94; TRPM2−/−: 3.02 ± 0.44; TRPM2−/− PD: 4.79 ± 0.54; CPu: Male: TRPM2+/+: 2.77 ± 0.41, TRPM2+/+ PD: 21.08 ± 3.54; TRPM2+/−: 2.51 ± 0.33; TRPM2+/− PD: 3.74 ± 0.60; TRPM2−/−: 2.46 ± 0.28; TRPM2−/− PD: 6.63 ± 0.78; Female: TRPM2+/+: 2.86 ± 0.58; TRPM2+/+ PD: 21.77 ± 3.04; TRPM2+/−: 2.95 ± 0.85; TRPM2+/− PD: 6.52 ± 0.90; TRPM2−/−: 2.52 ± 0.26; TRPM2−/− PD: 4.16 ± 0.48).

### 3.6 TRPM2 Knockdown Alleviates Cell Death and Improves Cell Viability in Co‐Culture Submitted to a PD In Vitro Model

The data gathered here indicate that the TRPM2 channel knockout was able to diminish inflammation by reducing microglial density, alleviating morphological changes and cytokine production, and reducing CD68 staining. However, the TRPM2‐knockout mice used here present a global deletion, making it unclear whether that calcium channel has different roles if selectively ablated. We thus aimed to better understand TRPM2's contribution specifically in neurons and microglia in the PD scenario. To do that, an in vitro method was performed. The SH‐SY5Y human cell line was differentiated to induce a dopaminergic neuron‐like phenotype (Figure S1a). The HMC3 human microglial cell line was also used. To simplify, here we called the differentiated SH‐SY5Y cells neurons and the HMC3 cells microglia. TRPM2 expression was validated in each cell line through RT‐qPCR (data not shown). To induce the in vitro PD, we treated cells with 6‐OHDA. The neurotoxin was able to reduce neuronal viability and promote neuronal death while no effects were observed in microglia. This is expected as microglia do not express the dopamine transporter needed for the 6‐OHDA action (Bu et al. 2021). Thus, we included a conditioned media treatment in the experiments, as molecules released by neurons have been shown to modulate microglial activity (Peng et al. 2021); however, no effects were observed for either neurons or microglia (Figure S2a–f). Considering that the direct contact between cells might be needed, we proceeded to a co‐culture system. Three approaches were adopted: (1) neurons and microglia were co‐cultured and then the 6‐OHDA was added [(*N* + M) 6‐OHDA] (Figure S2g); (2) microglia were treated with 6‐OHDA and then added to neurons [N (M 6‐OHDA)] (Figure S2j); (3) neurons were treated with 6‐OHDA and then microglia were added [N 6‐OHDA (M)] (Figure S2m). Cell viability was reduced and cell death increased in the first and third approaches (Figure S2h,l,n,o). Only cell viability was reduced in the second approach (Figure S2k,l). The percentage of phagocytic microglia and the cell ratio were calculated in the immunocytochemistry images (Figure S3). All the approaches produced an increase in the percentage of phagocytic microglia when cells were treated with 6‐OHDA (Figure S3a–d). A higher number of microglia and a lower number of neurons were evidenced in the first and third approaches (Figure S3e–h). Representative images of the control condition and the 6‐OHDA condition are shown in Figure S3l,j, where it is possible to visualize microglia phagocyting neuronal debris in the 6‐OHDA‐treated conditions (βTubIII staining inside microglia). All these data indicate that, in our PD in vitro model, 6‐OHDA acts on neurons promoting their death and the 6‐OHDA‐induced apoptotic neurons enhance microglia phagocytic activity in the co‐culture condition.

**FIGURE 9:**
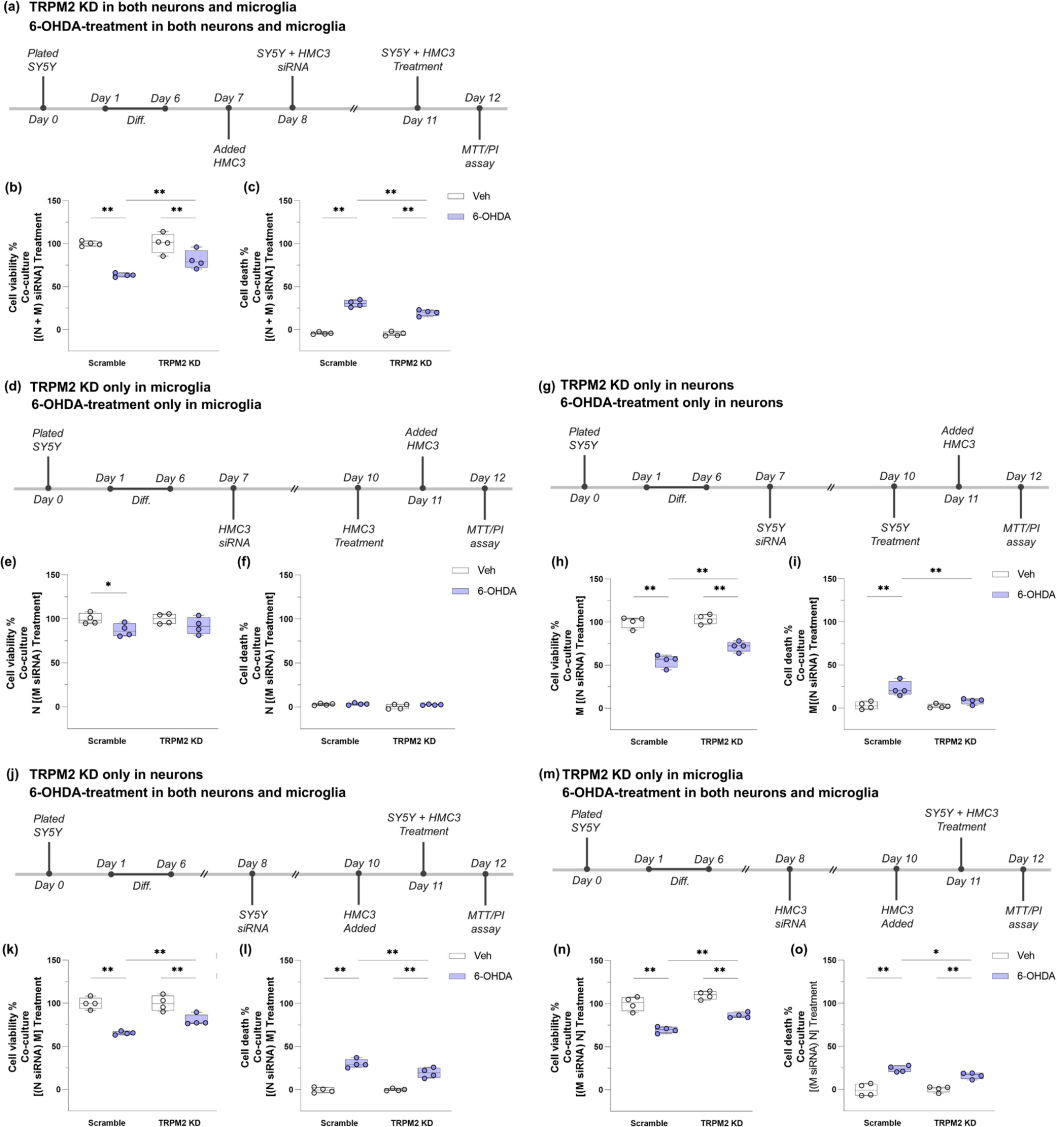
TRPM2 knockdown alleviate cell death in neuron–microglia co‐culture. Five different combinations of treatments were performed. (1) TRPM2 was knocked down in both neurons (N) and microglia (M) and then 6‐OHDA was added to both cells when in co‐culture—[(*N* + M) siRNA] Treatment. Timeline of experimental procedures (a), MTT assay (cell viability) (b), and PI assay (cell death) (c). (2) TRPM2 was knocked down only in microglia and then 6‐OHDA was added to microglia before adding them to the neurons—N [(M siRNA) Treatment]. Timeline of experimental procedures (d), MTT assay (cell viability) (e), and PI assay (cell death) (f). (3) TRPM2 was knocked down only in neurons and then 6‐OHDA was added to neurons before microglia was added—M [(N siRNA) Treatment]. Timeline of experimental procedures (g), MTT assay (cell viability) (h), and PI assay (cell death) (i). (4) TRPM2 was knocked down only in neurons, they were then added to the microglia, and 6‐OHDA was added to the co‐culture—[(N siRNA) M] Treatment. Timeline of experimental procedures (j), MTT assay (cell viability) (k), and PI assay (cell death) (l). (5) TRPM2 was knocked down only in microglia, then neurons were added, and 6‐OHDA was added to the co‐culture—[(M siRNA) N] Treatment. Timeline of experimental procedures (m), MTT assay (cell viability) (n), and PI assay (cell death) (o). Graphs show box plots with min/max whiskers and each dot represents the data from each analyzed replicate. Each condition has four independent replicates, and each independent replicate contains three technical repeats. **p < 0.01; *p < 0.05.

After having validated the PD in vitro model, we next assessed the TRPM2 involvement in different experimental approaches. In the single cell culture, TRPM2 knockdown was able to alleviate the decrease in neuronal viability and the increase in neuronal death (Figure S4). When in co‐culture, we selectively blocked neurons, microglia, or both cell TRPM2 channels to evaluate its specific contribution (Figure 9). Five approaches were adopted: (1) TRPM2 was silenced in both neurons and microglia and the 6‐OHDA‐treatment was performed in both neurons and microglia (Figure 9a–c); (2) TRPM2 was silenced only in microglia and the 6‐OHDA‐treatment was performed only in microglia (Figure 9d–f); (3) TRPM2 was silenced only in neurons and the 6‐OHDA‐treatment was performed only in neurons (Figure 9g–i); (4) TRPM2 was silenced only in neurons and the 6‐OHDA‐treatment was performed in both neurons and microglia (Figure 9j–l); and (5) TRPM2 was silenced only in microglia and the 6‐OHDA‐treatment was performed in both neurons and microglia (Figure 9m–o). An increase in cell viability and alleviation of cell death was observed in the first case {Cell viability: *siRNA* [F(1,12) = 5.098; *p* = 0.043]; *6‐OHDA* [F(1,12) = 47.668; *p* = 0.000]; cell death: *Interaction* [F(3,12) = 10.945; *p* = 0.006]}, third {Cell viability: *siRNA* [F(1,12) = 10.228; *p* = 0.008]; *6‐OHDA* [F(1,12) = 147.263; *p* = 0.000]; cell death: *Interaction* [F(3,12) = 6.654; *p* = 0.024]}, fourth {Cell viability: Interaction [F(3,12) = 5.166; *p* = 0.042]; cell death: *Interaction* [F(3,12) = 7.041; *p* = 0.021]}, and fifth approaches {Cell viability: *siRNA* [F(1,12) = 25.529; *p* = 0.000]; *6‐OHDA* [F(1,12) = 104.423; *p* = 0.000]; cell death: *6‐OHDA* [F(1,12) = 71.616; *p* = 0.000]}. These results lead us to three main interpretations: dying neurons are required to enhance microglial activity (6‐OHDA alone is not enough); neuronal TRPM2 is involved in the mechanism of 6‐OHDA‐induced cell death; and microglial TRPM2 may be involved in microglial activation; thus, inhibiting the channel before PD induction favors a more homeostatic microglial state, which alleviates the 6‐OHDA effect observed in the co‐culture.

### 3.7 TRPM2 Knockdown Reduced Phagocytosis and Inflammation‐Related Molecules Expression in Microglia

**FIGURE 10:**
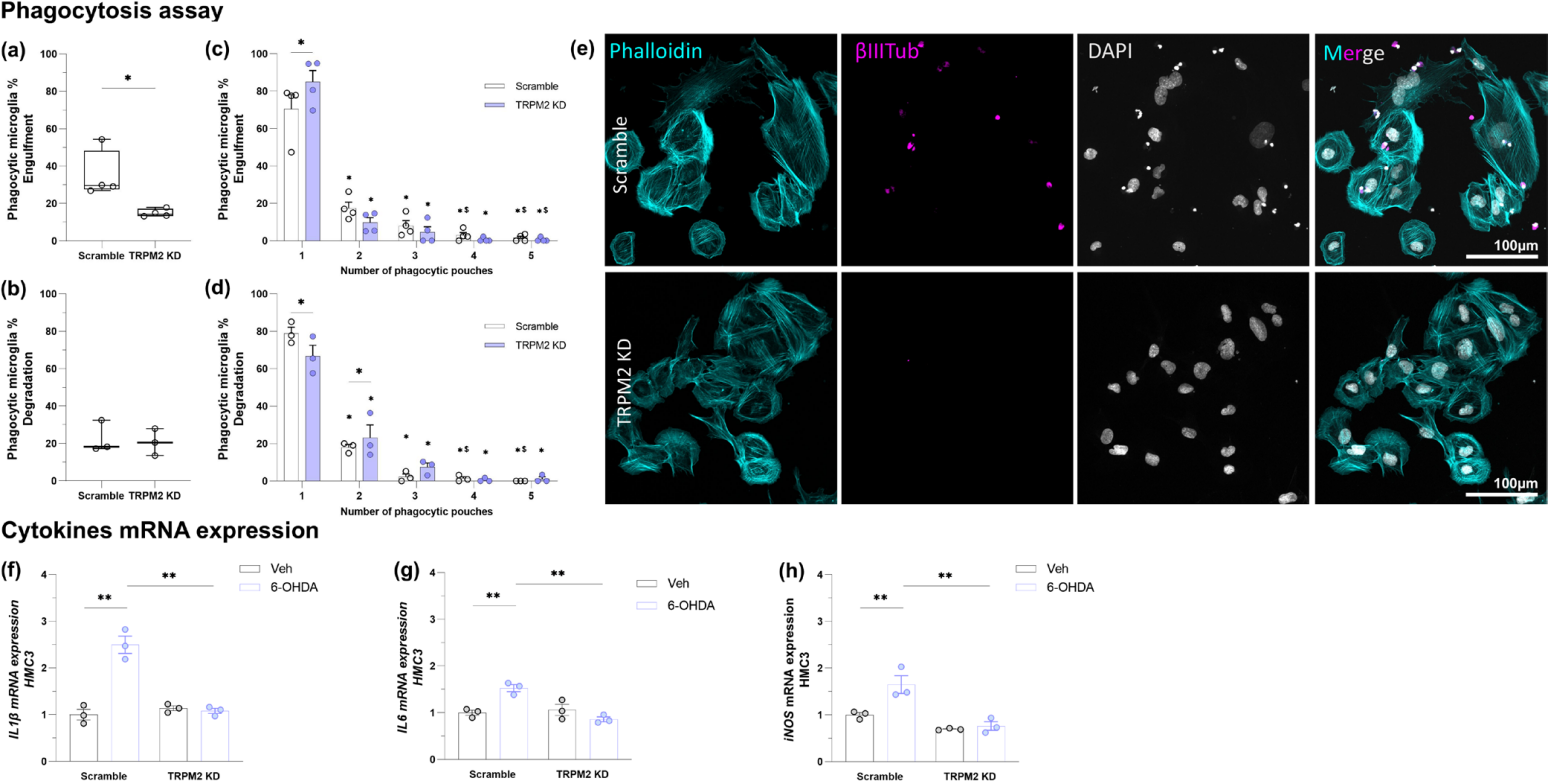
TRPM2 Knockdown reduced microglial activation and inflammation. Representative images of the phagocytosis assay (e). F‐actin was stained with Phalloidin (cyan), neurons were stained with β‐III‐Tubulin (βIIITub, purple), and nuclei are stained with DAPI (gray). Percentage of phagocytic microglia in the engulfment (a) and degradation (b). Percentage of phagocytic microglia with one, two, three, four, or five or more pouches in the engulfment (c) and degradation (d). IL‐1β mRNA expression (f), IL‐6 mRNA expression (g), and iNOS mRNA expression (h) of microglia. In (a) and (c), each condition has four independent replicates. In (b), (d), (f), (g), and (h), each condition has three independent replicates. Graphs show box plots with min/max whiskers or bars with mean and SEM and each dot represents the data from each analyzed replicate. *Comparison of number of phagocytic microglia with one phagocytic pouch with 2, 3, 4, or 5 pouches. ^$^Comparison of number of phagocytic microglia with two phagocytic pouches with 4 or 5 pouches. **p < 0.01; *p < 0.05; ^$^ *p* < 0.05.

To further explore the specific role of TRPM2 in microglia activation, we performed a phagocytosis assay and assessed the profile of inflammatory molecules (Figure 10). In the phagocytosis assay, microglial function was evaluated in the process of engulfing neuronal debris, 1.5 h after microglia were fed with apoptotic neurons (Figure 10a), and degrading it, 3 h after removing the nonengulfed apoptotic neurons (Figure 10b). The percentage of phagocytic microglia was reduced in TRPM2 knockdown microglia cells in the engulfment phase [F(1,6) = 9.497; *p* = 0.022]. No difference was observed in the degradation [F(1,4) = 0.096; *p* = 0.773]. Microglia were also classified according to the number of phagocytic pouches (Figure 10c,d). We noted that TRPM2 knockdown increased the number of phagocytic microglia with one phagocytic pouch in the engulfment phase (*p* = 0.014), and slightly reduced the ones with two, three, or four pouches. In addition, TRPM2 depletion decreases the number of phagocytic microglia with one phagocytic pouch (*p* = 0.016) while increasing the ones with two phagocytic pouches (*p* = 0.049) in the degradation phase. These results suggest that TRPM2 is important for microglial phagocytic activity.

To analyze the microglial inflammatory response, we evaluated IL‐1β (Figure 10f), IL‐6 (Figure 10g), and iNOS (Figure 10h) mRNA levels after 6‐OHDA treatment. We observed an increase in mRNA expression of all molecules in the control 6‐OHDA‐treated microglia {IL‐1β: *Interaction* [F(3,8) = 45.988; *p* = 0.000]; IL‐6: *Interaction* [F(3,8) = 20.993; *p* = 0.002]; iNOS: *Interaction* [F(3,8) = 7.107; *p* = 0.029]}. In addition, TRPM2 knocked down was able to reduce IL‐1β (*p* < 0.0001), IL‐6 (*p* < 0.0001), and iNOS (*p* < 0.0001) mRNA expression found enhanced after 6‐OHDA treatment. Taken together, these findings indicate that TRPM2 is important for microglial function (IL‐1β: Scramble Veh: 1.000 ± 0.113, Scramble 6‐OHDA: 2.496 ± 0.184, TRPM2 KD Veh: 1.137 ± 0.051, TRPM2 KD 6‐OHDA: 1.080 ± 0.056; IL‐6: Scramble Veh: 1.000 ± 0.054, Scramble 6‐OHDA: 1.528 ± 0.075, TRPM2 KD Veh: 1.059 ± 0.119, TRPM2 KD 6‐OHDA: 0.860 ± 0.050; iNOS: Scramble Veh: 1.000 ± 0.049, Scramble 6‐OHDA: 1.649 ± 0.191, TRPM2 KD Veh: 0.693 ± 0.012; TRPM2 KD 6‐OHDA: 0.762 ± 0.092).

## 4 Discussion

In this study, we used in vivo and in vitro approaches to better understand the TRPM2 channel involvement in the inflammatory response of PD, with a focus on microglial cells. We demonstrated that TRPM2 deletion or knockdown: (1) reduced dopaminergic neuronal death; (2) prevented increases in microglial and CD68 density; (3) reversed microglial morphological alterations observed in the wild‐type PD animals; (4) decreased levels of pro‐inflammatory cytokines; (5) prevented CD68‐area increase in microglia; (6) alleviated cell death and improved cell viability in a neuron–microglia co‐culture; and (7) reduced phagocytosis and inflammation‐related molecules expression in microglia. These results demonstrate that the TRPM2 plays a critical role in modulating microglial activation and inflammation in the 6‐OHDA model, highlighting TRPM2 as an emergent, potential therapeutical target for PD.

Others have reported protective effects of TRPM2 deletion in various neurological diseases. For instance, reduced demyelination, synapse loss, microglial activation, and pro‐inflammatory cytokines production were noted on TRPM2‐knockout mice induced to a model of multiple sclerosis (Shao et al. 2021). TRPM2−/− mice showed reduced pro‐inflammatory cytokine expression and microglial cell count in a temporal lobe epilepsy model (Hu et al. 2020). In the present study, we observed that TRPM2 deletion is neuroprotective in the PD mouse model, as the nigral dopaminergic cell death was alleviated in both male and female mice induced to the 6‐OHDA model. In addition, while both partial (TRPM2+/−) and complete (TRPM2−/−) deletions led to larger neuronal preservation compared to wild‐type (TRPM2+/+) PD mice, we noted that the complete deletion offered more neuroprotection. These findings align with our recent work, that showed neuroprotective effects of TRPM2 deletion by protecting against dopaminergic neuronal death and alleviating motor impairment as assessed by three different behavioral tests (Ferreira, Ulrich, Mori, et al. 2024b), and corroborate with previous studies of our group (Ferreira et al. 2022; Ferreira, Ulrich, Feng, et al. 2024a) and of others (Tamura et al. 2022; Vaidya et al. 2022; Ye et al. 2023) that used other PD models or pharmacological inhibitors.

In an effort to better understand TRPM2 role in PD, we decided to focus on the inflammatory aspect of the disease, in which microglia is a central protagonist. We demonstrated that microglial density, morphology alterations and function, as well as the cytokine profile, suggest an exacerbated inflammatory response after PD induction, which was not present in the partial or complete TRPM2 deletion mice. Overall, no difference was observed between partial and complete channel deletion, with both groups showing similar relief of inflammation. In addition, the beneficial effects were observed in both males and female animals. The use of female mice in research has been considerably increased over the past few years, as understanding the mechanisms involved in the pathology in both sexes may improve diagnosis and treatment strategies (Becegato and Silva 2024). In ischemic models, TRPM2 inhibition has shown controversial effects in males and females, as some studies evidenced only protection in males (Nakayama et al. 2013; Shimizu et al. 2013, 2016b; Shimizu et al. 2016a), while others showed neuroprotection in both sexes (Dietz et al. 2021; Jia et al. 2011). To our knowledge, no other group has included female animals when evaluating the role of TRPM2 in PD. Here we evidenced that TRPM2 deletion alleviated inflammation in both sexes, meaning that it could potentially represent a therapeutical target for men and women who suffer from PD.

Although the prevalence of PD is nearly twice as high in men than in women, with a male‐to‐female ratio of 1.81 (Feigin et al. 2019), very few studies consider sex as a variable. The inclusion of both sexes in studies is extremely important as it allows researchers to identify similar and/or divergent pathways that can better guide the development of more assertive treatment approaches. Our data indicate that female mice exhibit, to some extent, lower inflammation than males, as reduced microglia and CD68 density, more ramified microglia, and decreased levels of IL‐1α and IL‐6 were all noted in females. These alterations were observed despite the fact that no sex‐based difference was seen in neuronal loss. Some studies have reported a larger dopaminergic cell loss in female mice induced to different PD models (Gillies et al. 2014; Isenbrandt et al. 2023; Murray et al. 2003). However, this effect appears to be dependent on the toxin dose, animal strain, and estrous cycle phase, with higher cell loss observed when the model is induced in diestrus—lower estrogen levels—as is the case of the present study (Bourque et al. 2023; Gillies et al. 2014). Sex differences in the inflammatory profile were also observed by others when comparing male and female PD animals (Bourque et al. 2023; Isenbrandt et al. 2023). These differences may be attributed to the distinct transcriptional profiles of microglia in the midbrain and CPu between males and females, with immune‐related pathways being more pronounced in male microglia, suggesting a more inflammatory phenotype (Barko et al. 2022).

As our focus was to get a better picture of the TRPM2 channel in PD‐induced microglia activation, we extracted different variables as indicators of microglial activation. We found that animals from the PD wild‐type group showed increased microglial and CD68 density. These microglial cells have fewer endpoints and shorter branches, as well as higher cell compaction, reduced circularity, and lower area and perimeter, indicating morphological changes. In addition, cytokine levels, IFNγ, IL‐1α, IL‐1β, IL‐6, and TNFα were found to be increased in PD‐induced animals. As a measure of each microglial activation, the CD68 area was also evaluated in each cell, and a larger area was noted in TRPM2+/+ PD mice, suggesting an increase in microglial phagocytic activity. All these aspects are compatible with more reactive microglia and an enhanced inflamed environment. Interestingly, our results pointed out that TRPM2 ablation lessens the PD‐induced proinflammatory response in both sexes and brain regions. Combining the information from morphological classification, CD68 labeling, and cytokine levels, our data indicate that microglia from the PD wild‐type group have a reactive pro‐inflammatory phenotype and the ones from the control and TRPM2 knockout groups have a homeostatic‐like phenotype. It is important to stress that microglia is a complex cell, and although we tried to analyze molecular and morphological aspects, our study presents limitations and other techniques can be employed in the future to corroborate our data, such as transcriptomic, epigenetic, or proteomic analysis (Paolicelli et al. 2022).

Nevertheless, it is not clear if the TRPM2 from microglia or from neurons plays different roles in PD pathology. Recently, using a conditional deletion model, microglial TRPM2 was specifically deleted, and animals showed attenuated glial activation, inflammatory cytokine release, and epileptogenesis (Chen et al. 2023). In the same paper, when TRPM2 knockout was restricted to astrocytes, no effect was observed (Chen et al. 2023). In another study, TRPM2 was specifically deleted in neurons, and authors reported reduced brain infarct volume and neuronal death in an ischemic mouse model (Zong et al. 2022), but they did not evaluate neuroinflammation. Thus, it seems that TRPM2 deletion in either microglia or neurons contributes to an alleviation in the pathology of different neurological diseases. To better understand the intricate mechanism involved, we also conducted an in vitro approach in which human cell lines were used. Despite animal models being very useful in mimicking many pathological signals, there are intrinsic differences when comparing mouse and human microglia (Masuda et al. 2019), making it important to validate results in human models.

In our in vitro approach, we knocked down TRPM2 selectively in neurons, microglia, or both cells and then co‐cultured these cells. After treating the co‐culture with 6‐OHDA, similar effects were observed from microglia‐specific, neuron‐specific, or general TRPM2 knockdown, as all approaches increased cell viability and reduced cell death. We also treated microglia‐specific and neuron‐specific knockdowns with 6‐OHDA before co‐culturing them. While in the neuron‐specific knockdown, increased cell viability and reduced cell death were found, no robust effect was noted when 6‐OHDA treatment was only in microglia cells, which indicates that 6‐OHDA alone is not enough to fully activate microglia. When working with cultures, one thing that we need to have in mind is that the in vitro approach does not recapitulate all the complexity that is found in vivo. The establishment of co‐cultures includes the crosstalk between cells, better mimicking the complex interactions between cells, and creating more stable cultures (Goers et al. 2014). For instance, using the same cell lines as the present study, it was reported that tunneling nanotubes are formed as a way of communication between microglia and neurons, and these allow material transfer between cells that help maintain neuronal health (Chakraborty et al. 2023). We suggest that the presence of these communications might explain our observations, as by improving the resilience of one cell type (by TRPM2 inhibition) the other type also benefited from that.

Finally, our data indicate that TRPM2 knockdown reduced microglia phagocytic activity and inflammatory profile (reduced IL‐1β, IL‐6, and iNOS expression levels). TRPM2's contribution to microglial function was described before by others. The channel mediates calcium signals and ion currents (Kraft et al. 2004), morphological changes, production and release of pro‐inflammatory factors after stimulation (Raghunatha et al. 2020), cell activation, and generation of TNFα in microglial cells (Mortadza et al. 2018). Our study corroborates these data and reinforces the idea of a TRPM2 role in microglial function. It also highlights TRPM2 inhibition as a tool to modulate inflammation and microglial activation in CNS diseases. In PD, microglia from the midbrain actively participate in the phagocytosis of dying dopaminergic neurons bodies (Basurco et al. 2023), which can be beneficial. However, degenerating neurons may be phagocytized prematurely or excessively by microglia, aggravating the disease pathology (Butler et al. 2021). Thus, TRPM2 inhibition emerges as a tool to modulate microglia phagocytic activity and possibly alleviate the progression of PD.

We believe that TRPM2 may act through different mechanisms in microglia and neurons, but converge as the channel functions as a sensor of oxidative stress in both cell types. In microglia, intracellular calcium elevations serve as a key signaling event that triggers a range of microglial responses, including morphological remodeling, alterations in gene expression, production and release of cytokines and neurotrophic factors, upregulation of inflammatory markers, as well as increased proliferation, migration, and phagocytic activity (Pan and Garaschuk 2023). Changes in morphology, ramification, retraction, and migration appear to be mediated by P2Y receptors (Davalos et al. 2005; Kyrargyri et al. 2020; Milior et al. 2020). These receptors activate signaling pathways involving Akt, which in turn promote actin cytoskeleton reorganization and the expression and activation of integrins, key regulators of structural and morphological dynamics in microglia (Madry and Attwell 2015; Ohsawa et al. 2010). In neurons, TRPM2 contributes to cell death, particularly under pathological conditions. TRPM2‐mediated increases in intracellular calcium have been mechanistically linked to multiple downstream signaling pathways. Notably, we and others have demonstrated that inhibition of TRPM2 positively regulates the pro‐survival Akt/GSK/caspase‐3 signaling axis (Ferreira et al. 2022; Ferreira, Ulrich, Feng, et al. 2024a; Huang et al. 2017; Li et al. 2019). Additionally, TRPM2 has been shown to promote activation of the Cdk5/p25 complex via calpain‐dependent mechanisms, which lead to phosphorylation and inhibition of a key cellular peroxiredoxin involved in ROS detoxification, resulting in oxidative stress accumulation (Ko et al. 2019). Mitochondrial dysfunction represents another TRPM2‐linked neurotoxic mechanism, as TRPM2 activation has been implicated in promoting mitochondrial hyperfusion and triggering permeability transition through an Mfn2/Bcl‐1‐mediated pathway (Ye et al. 2023). These TRPM2‐mediated mechanisms ultimately culminate in neuronal death, which can in turn further activate microglia and amplify neuroinflammatory responses. To attribute specific phenotypes to microglial or neuronal TRPM2, it is essential to validate these findings using cell‐specific conditional knockout models. This will certainly help future studies into a better understanding of the specific TRPM2 contribution in PD. Nevertheless, as this study primarily focuses on the neuroinflammatory and microglial components, the findings gathered here suggest that TRPM2 plays an important role in the process.

While the results of this work provide evidence to support the immunomodulatory role of the TRPM2 in PD, several limitations should be acknowledged. First, the relatively small sample size in our in vivo experiments represents a limitation. However, the post hoc power analysis demonstrated high statistical power (> 80%) for the majority of key findings. This allowed us to identify meaningful effects with minimal animal use, in alignment with ethical research principles. Nonetheless, future studies with an increased sample size are needed and will reinforce the robustness of our findings. Second, the in vitro part of the study relied on immortalized cell lines. They present modifications and diverge in physiological aspects from in vivo and primary cells (Timmerman et al. 2018; Xicoy et al. 2017). However, they are accessible, reproducible, and suitable for initial mechanistic investigations and helped to confirm the translational relevance of TRPM2 in microglial activation and cell death in a human cell condition. Future studies could use either primary microglia or iPSC‐derived cells, and also specific TRPM2 microglia‐knockout to delve into the underlying mechanisms that account for the beneficial effects of TRPM2 deletion observed here. Finally, while microglial morphology offers important clues regarding functional state, it is not always a direct indicator of function. To strengthen our interpretations, we combined morphological assessment with functional (CD68 expression) and molecular (cytokine profiling) analyses. Nevertheless, including genomic and proteomic profiling in future studies will be essential to characterize microglial responses and clarify the specific roles of TRPM2 in regulating microglial function in PD.

In summary, we extend our previous findings that TRPM2 is neuroprotective in the 6‐OHDA model of PD and showed that it also prevents the inflammation and microglial reactivation that is present in that PD model. TRPM2 inhibition reduced microglia density and phagocytic activity, restored its morphology, and reduced pro‐inflammatory cytokine levels after 6‐OHDA‐injection. We demonstrated that these effects are observable in both male and female animals. Additionally, partial and complete knockout mice had, generally, similar protective benefits. These results were corroborated in vitro, showing that TRPM2 protects dopaminergic‐like neurons and microglia co‐culture against 6‐OHDA. Lastly, we showed that TRPM2 is involved in microglia phagocytosis and inflammatory cytokine expression. Overall, the present study points to TRPM2 as a potential pharmacological target for PD by targeting the associated neuroinflammation.

## Author Contributions

A.F.F.F. and L.R.G.B. conceived the research and designed the experiments. A.F.F.F. performed the experiments and analyzed the data. L.R.G.B. contributed to the project administration, funding acquisition, supervision, and writing. Z.P.F. and H.S.S. contributed to the conceptualization, intellectual input, funding, and writing. A.F.F.F. wrote the original draft. All authors read, reviewed, and approved the final version of the manuscript.

## Ethics Statement

Animal experiments were approved by the Ethics Committee for Animal Research of the Institute of Biomedical Sciences of the University of São Paulo (CEUA‐ICB/USP, Brazil, protocol number: 8395080450).

## Consent

The authors have nothing to report.

## Conflicts of Interest

The authors declare no conflicts of interest.

## Supporting information


**Figure S1.** Supporting Figure.

**Figure S2.** Supporting Figure.

**Figure S3.** Supporting Figure.

**Figure S4.** Supporting Figure.

**Table S1.** Summary data of Skeleton analysis.
**Table S2** Summary data of Fractal analysis.
**Table S3** Summary data of cytokine levels assessed by ELISA.
**Table S4** Statistical power calculation (in percentage) of in vivo data.

## Data Availability

The data that support the findings of this study are available from the corresponding author upon reasonable request.
