# Supplementary figures and images for: Microglial Activation and Inflammatory Responses in Parkinson's Disease Models Are Attenuated by TRPM2 Depletion
Source: Glia. 2025 Jul 15;73(10):2035–56. doi: 10.1002/glia.70055 (PMC12334870; doi:10.1002/glia.70055)

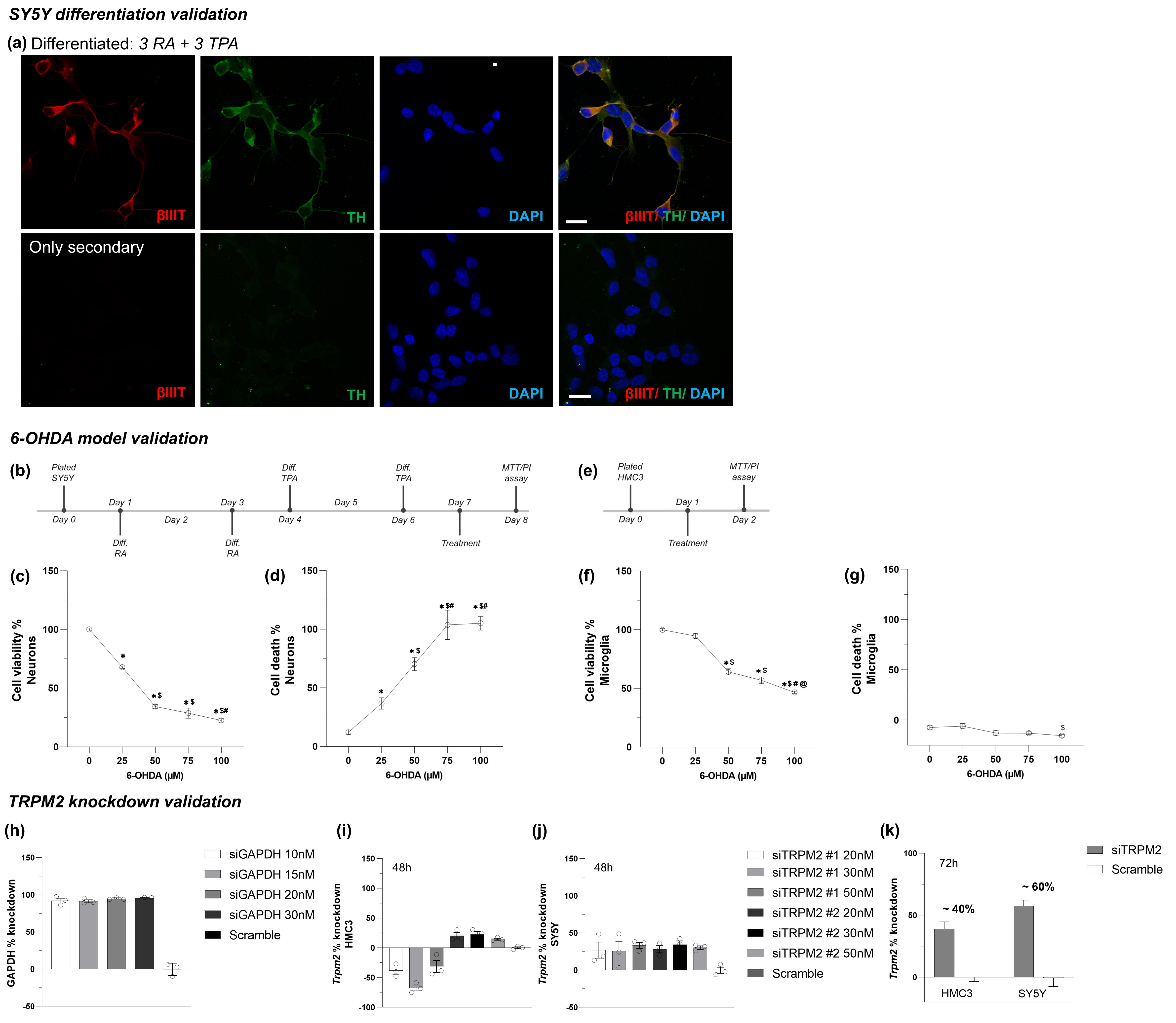

Supplement: Supplementary file 1 — Figure S1. Supporting Figure. [file GLIA-73-2035-s001.tif]

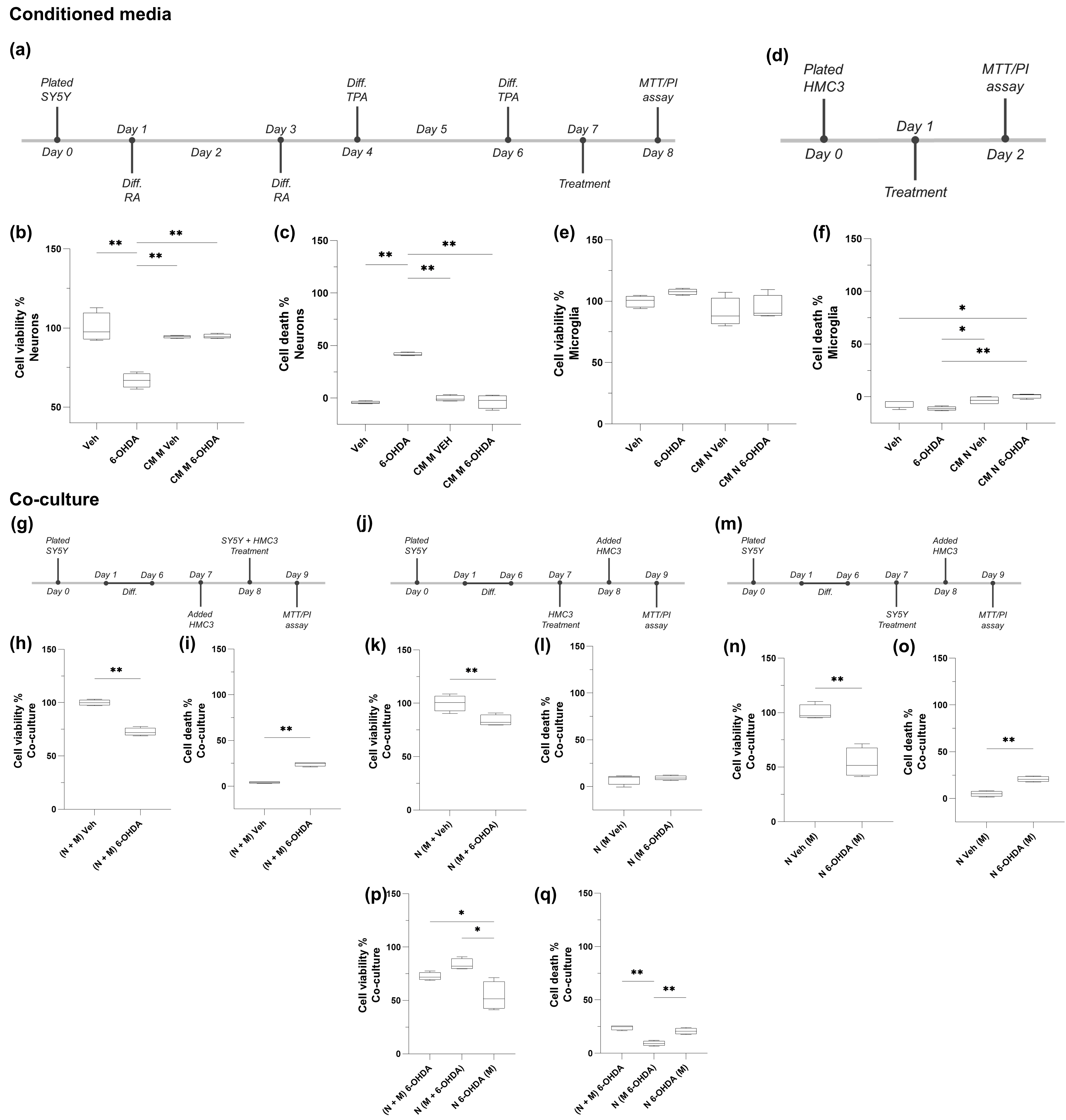

Supplement: Supplementary file 2 — Figure S2. Supporting Figure. [file GLIA-73-2035-s005.tif]

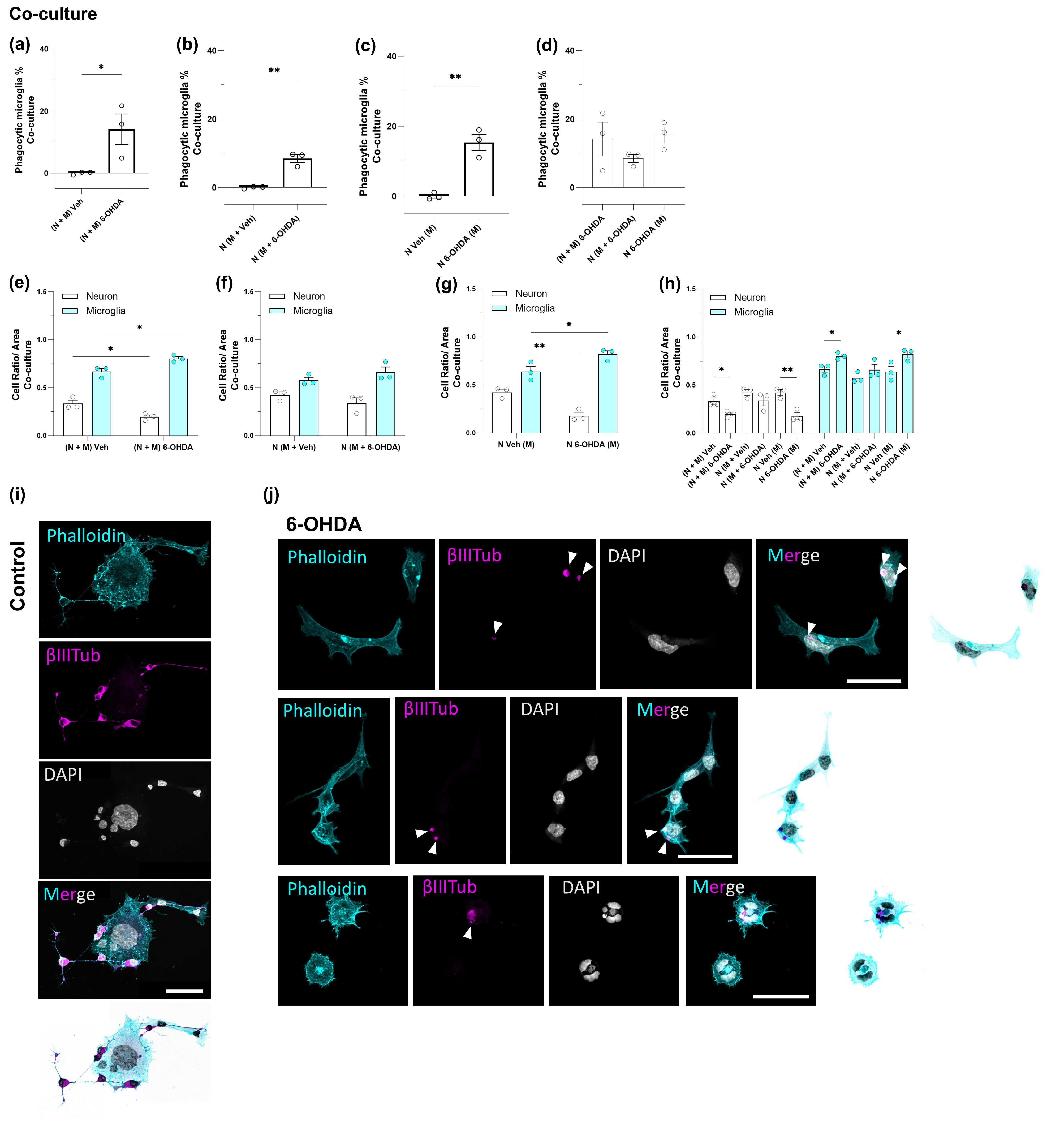

Supplement: Supplementary file 3 — Figure S3. Supporting Figure. [file GLIA-73-2035-s004.tif]

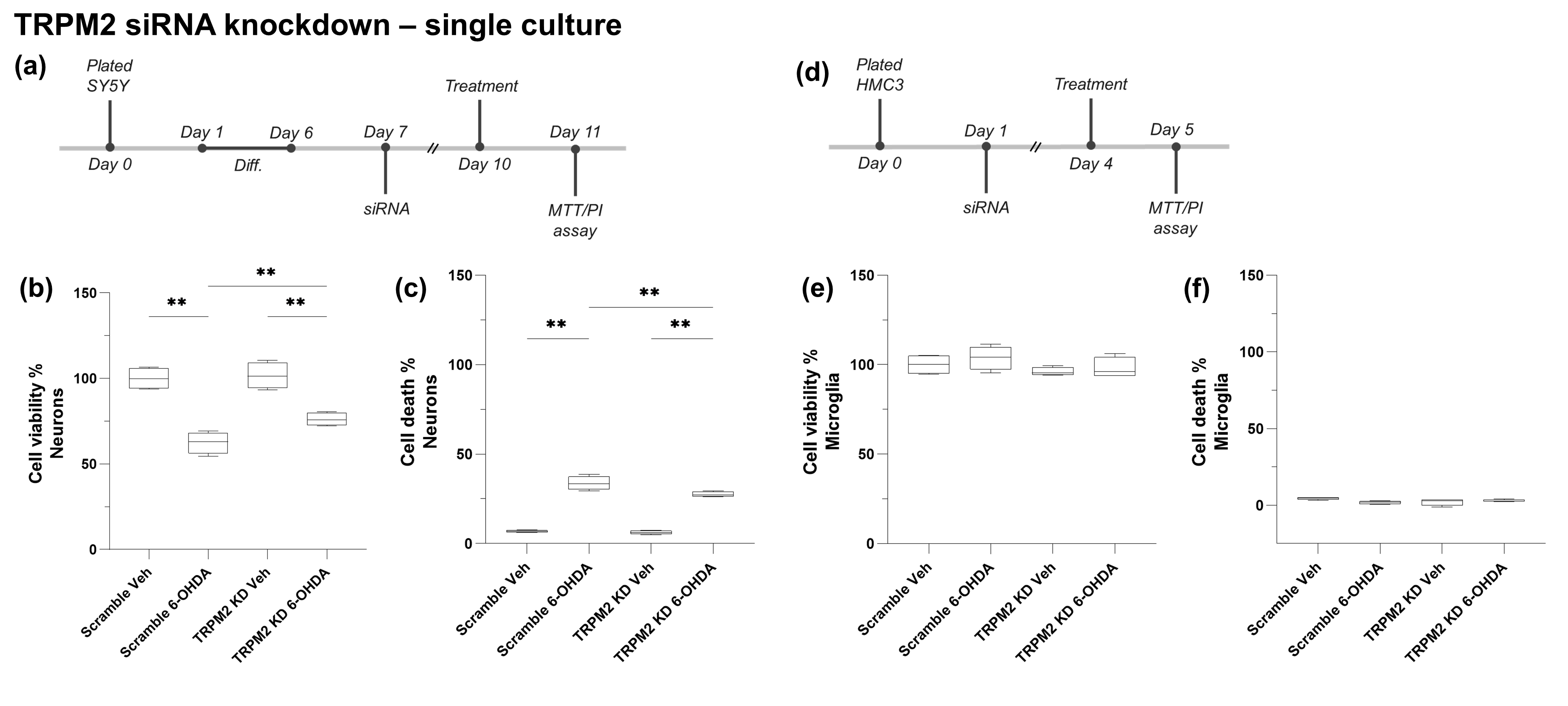

Supplement: Supplementary file 4 — Figure S4. Supporting Figure. [file GLIA-73-2035-s002.tif]
