# Supplementary material for: Microglial Activation and Inflammatory Responses in Parkinson's Disease Models Are Attenuated by TRPM2 Depletion
Source: Glia. 2025 Jul 15;73(10):2035–56. doi: 10.1002/glia.70055 (PMC12334870; doi:10.1002/glia.70055)
Supplement: Supplementary file 5 — Table S1. Summary data of Skeleton analysis. Table S2 Summary data of Fractal analysis. Table S3 Summary data of cytokine levels assessed by ELISA. Table S4 Statistical power calculation (in percentage) of in vivo data. [file GLIA-73-2035-s003.docx]

**Table S1:** Summary data of Skeleton analysis.

|  |  | **TRPM2+/+** | **TRPM2+/+ PD** | **TRPM2+/-** | **TRPM2+/- PD** | **TRPM2 -/-** | **TRPM2 -/- PD** |
| --- | --- | --- | --- | --- | --- | --- | --- |
| **Substantia nigra** | | **Mean±SEM** | **Mean±SEM** | **Mean±SEM** | **Mean±SEM** | **Mean±SEM** | **Mean±SEM** |
| **Males** | **Endpoint** | 49.87±2.20 | 31.76±2.28 | 52.88±7.14 | 48.38±3.06 | 47.38±3.06 | 53.22±4.38 |
|  | **Branch length** | 262.89±23.33 | 163.23±11.81 | 323.26±30.05 | 273.20±24.33 | 289.47±30.79 | 303.30±32.88 |
| **Females** | **Endpoint** | 78.46±7.91 | 51.12±1.75 | 82.65±5.49 | 78.50±3.82 | 75.94±7.73 | 77.38±1.35 |
|  | **Branch length** | 447.36±41.58 | 281.42±31.23 | 475.44±41.53 | 395.17±34.25 | 405.10±17.95 | 414.43±13.07 |
| **Striatum** | |  |  |  |  |  |  |
| **Males** | **Endpoint** | 71.06±1.33 | 44.44±2.39 | 75.48±6.49 | 72.85±9.51 | 86.18±3.02 | 65.88±1.42 |
|  | **Branch length** | 406.81±13.74 | 247.74±4.90 | 477.46±58.56 | 399.54±46.92 | 494.62±34.25 | 389.79±27.87 |
| **Females** | **Endpoint** | 89.08±6.31 | 59.47±1.35 | 91.61±6.44 | 76.06±2.95 | 78.65±8.09 | 79.49±3.21 |
|  | **Branch length** | 482.85±43.46 | 345.43±39.59 | 465.08±15.63 | 430.63±31.45 | 451.32±23.52 | 468.04±17.79 |

**Table S2:** Summary data of Fractal analysis.

|  |  | **TRPM2+/+** | | **TRPM2+/+ PD** | | **TRPM2+/-** | | **TRPM2+/- PD** | | **TRPM2 -/-** | | **TRPM2 -/- PD** | |  |
| --- | --- | --- | --- | --- | --- | --- | --- | --- | --- | --- | --- | --- | --- | --- |
| **Substantia nigra** | | **Mean±SEM** | | **Mean±SEM** | | **Mean±SEM** | | **Mean±SEM** | | **Mean±SEM** | | **Mean±SEM** | |  |
| **Male** | **Fractal dimension** | 1.466±0.013 | | 1.436±0.011 | | 1.463±0.016 | | 1.452±0.013 | | 1.490±0.013 | | 1.470±0.013 | |  |
|  | **Lacunarity** | 0.384±0.009 | | 0.389±0.011 | | 0.397±0.006 | | 0.393±0.007 | | 0.381±0.009 | | 0.403±0.005 | |  |
|  | **Density** | 0.072±0.003 | | 0.094±0.003 | | 0.072±0.003 | | 0.076±0.003 | | 0.072±0.002 | | 0.076±0.003 | |  |
|  | **Span ratio** | 1.412±0.065 | | 1.811±0.139 | | 1.527±0.075 | | 1.668±0.111 | | 1.426±0.038 | | 1.531±0.061 | |  |
|  | **Circularity** | 0.837±0.012 | | 0.762±0.021 | | 0.809±0.015 | | 0.793±0.019 | | 0.837±0.006 | | 0.808±0.010 | |  |
|  | **Maximum Span Across Hull** | 399.42±20.24 | | 237.01±10.37 | | 430.02±12.98 | | 361.42±14.65 | | 449.74±20.28 | | 400.28±15.66 | |  |
|  | **Area** | 5788.51±448.19 | | 1652.17±121.24 | | 6187.82±403.63 | | 4372.26±452.93 | | 7333.95±686.47 | | 5416.72±433.55 | |  |
|  | **Perimeter** | 290.89±12.79 | | 162.82±6.20 | | 306.83±9.15 | | 255.60±11.10 | | 323.85±15.08 | | 283.56±10.91 | |  |
|  | **Width of Bounding Rectangle** | 335.35±14.82 | | 197.48±9.75 | | 358.85±15.34 | | 310.70±15.34 | | 377.38±14.40 | | 354.40±15.98 | |  |
|  | **Height of bounding rectangle** | 329.00±18.14 | | 189.60±9.56 | | 360.00±14.53 | | 292.48±14.04 | | 362.76±20.79 | | 313.72±12.27 | |  |
|  | **Maximum Radius from Hull's centre of mass** | 216.65±10.39 | | 128.34±5.78 | | 234.40±6.74 | | 197.13±7.60 | | 244.12±12.49 | | 216.31±8.32 | |  |
|  | **Max/Min radii** | 1.65±0.06 | | 2.07±0.15 | | 1.79±0.08 | | 1.89±0.11 | | 1.65±0.04 | | 1.72±0.07 | |  |
|  | **CV for all Radii** | | 0.14±0.01 | | 0.20±0.01 | | 0.17±0.01 | | 0.18±0.01 | | 0.15±0.01 | | 0.16±0.01 | |
|  | **Mean Radius** | | 178.80±8.16 | | 102.30±4.09 | | 189.36±6.13 | | 159.51±6.62 | | 197.31±9.45 | | 177.72±7.29 | |
|  | **Diameter of bounding circle** | | 402.87±20.20 | | 23870±10.32 | | 434.67±12.86 | | 365.07±14.50 | | 451.05±20.38 | | 403.96±15.54 | |
|  | **Maximum radius from circle's centre** | | 201.43±10.10 | | 119.35±5.16 | | 217.34±6.43 | | 182.54±7.25 | | 225.53±10.19 | | 201.98±7.77 | |
|  | **Max/min radii from Circle's Centre** | | 1.63±0.06 | | 2.30±0.24 | | 1.85±0.12 | | 1.86±0.12 | | 1.66±0.07 | | 1.74±0.14 | |
|  | **CV for all Radii from circle's centre** | | 0.13±0.01 | | 0.18±0.01 | | 0.15±0.01 | | 0.15±0.01 | | 0.14±0.01 | | 0.13±0.0 | |
|  | **Mean Radius from Circle's Centre** | | 180.52±8.13 | | 103.17±4.16 | | 191.54±5.95 | | 161.92±6.67 | | 199.87±9.44 | | 181.28±7.42 | |
| **Female** | **Fractal dimension** | | 1.485±0.010 | | 1.446±0.012 | | 1.479±0.015 | | 1.500±0.007 | | 1.465±0.012 | | 1.498±0.011 | |
|  | **Lacunarity** | | 0.397±0.009 | | 0.380±0.007 | | 0.386±0.010 | | 0.402±0.010 | | 0.384±0.007 | | 0.401±0.014 | |
|  | **Density** | | 0.074±0.003 | | 0.090±0.004 | | 0.074±0.003 | | 0.081±0.003 | | 0.072±0.003 | | 0.078±0.003 | |
|  | **Span ratio** | | 1.365±0.070 | | 1.987±0.140 | | 1.421±0.088 | | 1.553±0.070 | | 1.370±0.049 | | 1.537±0.089 | |
|  | **Circularity** | | 0.859±0.015 | | 0.741±0.021 | | 0.821±0.013 | | 0.816±0.013 | | 0.843±0.010 | | 0.813±0.015 | |
|  | **Maximum Span Across Hull** | | 405.63±14.54 | | 290.92±17.81 | | 386.08±10.85 | | 369.22±14.66 | | 403.65±13.18 | | 403.72±12.54 | |
|  | **Area** | | 5995.16±396.81 | | 2398.17±276.76 | | 5235.99±377.50 | | 4656.12±374.93 | | 6056.49±437.80 | | 5455.40±426.93 | |
|  | **Perimeter** | | 294.01±10.51 | | 196.02±10.74 | | 280.04±9.26 | | 261.94±10.51 | | 296.57±10.69 | | 286.47±11.21 | |
|  | **Width of Bounding Rectangle** | | 352.73±12.52 | | 255.20±18.72 | | 328.88±11.12 | | 322.96±14.18 | | 350.00±12.21 | | 347.16±16.61 | |
|  | **Height of bounding rectangle** | | 340.27±13.16 | | 204.36±9.60 | | 326.31±12.20 | | 284.30±14.47 | | 337.00±13.06 | | 319.68±12.75 | |
|  | **Maximum Radius from Hull's centre of mass** | | 220.26±7.58 | | 158.36±9.58 | | 208.91±6.46 | | 201.19±8.47 | | 220.97±7.47 | | 219.89±6.37 | |
|  | **Max/Min radii** | | 1.63±0.06 | | 2.26±0.16 | | 1.72±0.07 | | 1.86±0.08 | | 1.63±0.07 | | 1.84±0.06 | |
|  | **CV for all Radii** | | 0.15±0.01 | | 0.20±0.02 | | 0.15±0.01 | | 0.18±0.01 | | 0.14±0.01 | | 0.18±0.01 | |
|  | **Mean Radius** | | 176.66±6.06 | | 125.47±7.50 | | 171.79±5.30 | | 161.29±6.43 | | 181.71±6.49 | | 176.79±5.57 | |
|  | **Diameter of bounding circle** | | 406.21±14.53 | | 292.17±17.21 | | 389.34±10.76 | | 371.26±14.67 | | 407.38±13.37 | | 408.95±13.00 | |
|  | **Maximum radius from circle's centre** | | 203.10±7.27 | | 146.09±8.86 | | 194.67±5.38 | | 185.63±7.34 | | 203.69±6.69 | | 204.47±6.50 | |
|  | **Max/min radii from Circle's Centre** | | 1.61±0.07 | | 2.070.14 | | 1.63±0.09 | | 1.87±0.10 | | 1.60±0.11 | | 1.75±0.08 | |
|  | **CV for all Radii from circle's centre** | | 0.14±0.01 | | 0.18±0.02 | | 0.14±0.01 | | 0.16±0.01 | | 0.12±0.01 | | 0.15±0.01 | |
|  | **Mean Radius from Circle's Centre** | | 179.48±5.93 | | 126.50±7.37 | | 173.32±5.41 | | 163.48±6.53 | | 184.29±6.18 | | 179.27±5.59 | |
| **Striatum** | | |  | |  | |  | |  | |  | |  | |
| **Male** | **Fractal dimension** | | 1.500±0.010 | | 1.554±0.013 | | 1.522±0.015 | | 1.535±0.012 | | 1.519±0.016 | | 1.529±0.014 | |
|  | **Lacunarity** | | 0.729±0.020 | | 0.766±0.029 | | 0.705±0.028 | | 0.719±0.029 | | 0.717±0.025 | | 0.733±0.032 | |
|  | **Density** | | 0.165±0.005 | | 0.374±0.016 | | 0.158±0.007 | | 0.196±0.010 | | 0.164±0.009 | | 0.189±0.010 | |
|  | **Span ratio** | | 1.438±0.045 | | 1.808±0.128 | | 1.538±0.049 | | 1.456±0.044 | | 1.377±0.063 | | 1.396±0.052 | |
|  | **Circularity** | | 0.839±0.011 | | 0.773±0.023 | | 0.822±0.009 | | 0.840±0.011 | | 0.856±0.013 | | 0.847±0.009 | |
|  | **Maximum Span Across Hull** | | 418.23±13.84 | | 275.04±10.90 | | 453.06±13.57 | | 397.75±11.09 | | 416.53±12.00 | | 393.06±12.94 | |
|  | **Area** | | 6289.034±326.134 | | 2240.554±190.586 | | 6899.113±347.670 | | 5708.689±421.958 | | 6591.217±378.263 | | 5630.594±356.856 | |
|  | **Perimeter** | | 305.454±8.593 | | 188.025±7.358 | | 322.781±7.887 | | 288.138±9.542 | | 308.629±7.841 | | 286.692±9.513 | |
|  | **Width of Bounding Rectangle** | | 372.20±12.84 | | 203.70±12.47 | | 403.79±14.84 | | 355.35±11.35 | | 362.70±12.93 | | 335.74±12.68 | |
|  | **Height of bounding rectangle** | | 350.55±12.35 | | 244.10±10.38 | | 344.74±13.91 | | 310.30±18.49 | | 358.40±11.07 | | 326.74±14.17 | |
|  | **Maximum Radius from Hull's centre of mass** | | 230.00±8.74 | | 150.08±7.00 | | 247.31±7.68 | | 218.06±5.44 | | 228.29±6.49 | | 218.37±8.98 | |
|  | **Max/Min radii** | | 1.726±0.074 | | 2.102±0.157 | | 1.781±0.056 | | 1.727±0.080 | | 1.704±0.078 | | 1.744±0.076 | |
|  | **CV for all Radii** | | 0.155±0.012 | | 0.205±0.015 | | 0.164±0.008 | | 0.158±0.010 | | 0.147±0.011 | | 0.162±0.012 | |
|  | **Mean Radius** | | 187.51±5.36 | | 115.41±4.57 | | 198.97±5.32 | | 175.49±5.62 | | 186.85±4.76 | | 173.02±5.92 | |
|  | **Diameter of bounding circle** | | 422.41±13.80 | | 276.71±10.97 | | 456.93±12.93 | | 400.76±11.25 | | 419.86±11.88 | | 396.51±12.93 | |
|  | **Maximum radius from circle's centre** | | 211.20±6.90 | | 138.35±5.49 | | 228.46±6.47 | | 200.38±5.62 | | 209.93±5.94 | | 198.25±6.47 | |
|  | **Max/min radii from Circle's Centre** | | 1.652±0.098 | | 1.999±0.148 | | 1.646±0.056 | | 1.582±0.061 | | 1.653±0.087 | | 1.625±0.080 | |
|  | **CV for all Radii from circle's centre** | | 0.127±0.010 | | 0.187±0.014 | | 0.137±0.008 | | 0.138±0.011 | | 0.127±0.011 | | 0.135±0.011 | |
|  | **Mean Radius from Circle's Centre** | | 189.84±5.69 | | 117.40±4.66 | | 201.17±4.94 | | 176.26±5.59 | | 188.51±4.65 | | 175.22±6.13 | |
| **Female** | **Fractal dimension** | | 1.566±0.016 | | 1.546±0.009 | | 1.560±0.014 | | 1.560±0.010 | | 1.528±0.007 | | 1.525±0.009 | |
|  | **Lacunarity** | | 0.662±0.026 | | 0.724±0.021 | | 0.630±0.018 | | 0.690±0.019 | | 0.669±0.019 | | 0.715±0.016 | |
|  | **Density** | | 0.215±0.018 | | 0.366±0.021 | | 0.205±0.011 | | 0.265±0.021 | | 0.183±0.007 | | 0.195±0.008 | |
|  | **Span ratio** | | 1.381±0.054 | | 1.611±0.071 | | 1.380±0.052 | | 1.391±0.042 | | 1.407±0.043 | | 1.372±0.055 | |
|  | **Circularity** | | 0.868±0.009 | | 0.806±0.014 | | 0.844±0.012 | | 0.845±0.008 | | 0.851±0.007 | | 0.850±0.008 | |
|  | **Maximum Span Across Hull** | | 412.86±14.04 | | 261.24±13.50 | | 437.27±15.12 | | 351.97±19.77 | | 429.17±11.61 | | 401.23±11.81 | |
|  | **Area** | | 6395.889±358.474 | | 2308.933±275.770 | | 7128.346±504.387 | | 4839.768±495.445 | | 6888.643±482.287 | | 6034.369±381.962 | |
|  | **Perimeter** | | 302.682±8.903 | | 184.003±9.835 | | 322.206±10.782 | | 261.864±14.170 | | 315.436±10.134 | | 295.291±9.535 | |
|  | **Width of Bounding Rectangle** | | 352.63±15.00 | | 212.65±13.48 | | 373.69±15.75 | | 327.56±21.09 | | 362.32±15.63 | | 357.00±10.98 | |
|  | **Height of bounding rectangle** | | 340.38±16.85 | | 215.55±13.93 | | 370.50±15.97 | | 290.33±15.65 | | 366.68±12.09 | | 348.38±14.15 | |
|  | **Maximum Radius from Hull's centre of mass** | | 229.35±6.78 | | 145.66±7.13 | | 238.58±8.06 | | 195.82±11.29 | | 234.79±6.22 | | 218.87±7.76 | |
|  | **Max/Min radii** | | 1.695±0.069 | | 1.897±0.104 | | 1.676±0.082 | | 1.720±0.056 | | 1.679±0.061 | | 1.598±0.053 | |
|  | **CV for all Radii** | | 0.147±0.012 | | 0.185±0.017 | | 0.144±0.013 | | 0.149±0.008 | | 0.146±0.010 | | 0.137±0.010 | |
|  | **Mean Radius** | | 183.53±5.46 | | 113.66±6.61 | | 196.55±6.47 | | 160.27±8.68 | | 193.42±6.11 | | 181.90±5.86 | |
|  | **Diameter of bounding circle** | | 415.58±13.78 | | 262.33±13.57 | | 442.35±14.89 | | 356.86±19.71 | | 431.91±12.84 | | 404.23±12.43 | |
|  | **Maximum radius from circle's centre** | | 207.79±6.89 | | 131.16±6.79 | | 221.17±7.44 | | 178.43±9.85 | | 215.95±6.42 | | 202.11±6.22 | |
|  | **Max/min radii from Circle's Centre** | | 1.630±0.080 | | 1.824±0.091 | | 1.617±0.090 | | 1.680±0.096 | | 1.552±0.076 | | 1.625±0.082 | |
|  | **CV for all Radii from circle's centre** | | 0.130±0.012 | | 0.160±0.011 | | 0.128±0.014 | | 0.128±0.011 | | 0.120±0.010 | | 0.119±0.011 | |
|  | **Mean Radius from Circle's Centre** | | 186.30±5.70 | | 114.77±6.52 | | 198.12±6.40 | | 162.47±8.94 | | 194.43±5.98 | | 183.44±6.06 | |

**Table S3:** Summary data of cytokine levels assessed by ELISA.

|  |  | **TRPM2+/+** | **TRPM2+/+ PD** | **TRPM2+/-** | **TRPM2+/- PD** | **TRPM2 -/-** | **TRPM2 -/- PD** |
| --- | --- | --- | --- | --- | --- | --- | --- |
| **Substantia Nigra** | | **Mean±SEM** | **Mean±SEM** | **Mean±SEM** | **Mean±SEM** | **Mean±SEM** | **Mean±SEM** |
| **Male** | **INFγ** | 0.717±0.043 | 0.774±0.029 | 0.664±0.007 | 0.662±0.049 | 0.730±0.052 | 0.692±0.044 |
|  | **IL1α** | 1.659±0.456 | 5.644±0.358 | 3.228±1.112 | 3.683±1.355 | 4.119±0.691 | 3.057±1.121 |
|  | **IL1β** | 2.697±0.191 | 3.502±0.132 | 2.667±0.111 | 2.494±0.204 | 2.861±0.156 | 2.594±0.323 |
|  | **IL6** | 2.694±0.155 | 3.241±0.144 | 1.978±0.066 | 2.791±0.130 | 2.564±0.154 | 2.106±0.242 |
|  | **IL10** | 0.576±0.069 | 0.488±0.028 | 0.435±0.029 | 0.494±0.050 | 0.512±0.033 | 0.476±0.044 |
|  | **TNFα** | 0.562±0.042 | 0.777±0.038 | 0.502±0.034 | 0.561±0.023 | 0.547±0.032 | 0.502±0.062 |
| **Female** | **INFγ** | 0.706±0.013 | 0.734±0.101 | 0.746±0.063 | 0.683±0.032 | 0.775±0.067 | 0.718±0.054 |
|  | **IL1α** | 3.567±0.997 | 2.583±0.772 | 3.322±0.406 | 1.494±0.168 | 1.542±0.142 | 1.670±0.549 |
|  | **IL1β** | 2.525±0.088 | 3.780±0.722 | 2.799±0.112 | 2.334±0.090 | 2.957±0.234 | 2.627±0.261 |
|  | **IL6** | 2.152±0.034 | 3.584±0.251 | 2.462±0.209 | 2.277±0.053 | 2.612±0.147 | 2.558±0.265 |
|  | **IL10** | 0.443±0.016 | 0.416±0.025 | 0.499±0.048 | 0.450±0.015 | 0.502±0.042 | 0.459±0.035 |
|  | **TNFα** | 0.490±0.033 | 0.818±0.179 | 0.575±0.047 | 0.494±0.030 | 0.622±0.086 | 0.573±0.050 |
| **Striatum** |  |  |  |  |  |  |  |
| **Male** | **INFγ** | 1.159±0.063 | 1.448±0.025 | 1.221±0.108 | 1.165±0.095 | 1.296±0.055 | 1.165±0.027 |
|  | **IL1α** | 1.466±0.182 | 2.819±0.659 | 1.418±0.387 | 1.439±0.084 | 1.334±0.121 | 1.520±0.198 |
|  | **IL1β** | 4.398±0.088 | 4.880±0.018 | 4.481±0.096 | 4.564±0.138 | 4.462±0.166 | 4.152±0.129 |
|  | **IL6** | 3.079±0.174 | 4.430±0.316 | 2.896±0.189 | 2.570±0.473 | 3.234±0.351 | 2.743±0.242 |
|  | **IL10** | 1.026±0.136 | 1.009±0.086 | 1.103±0.143 | 0.900±0.022 | 1.158±0.101 | 0.971±0.044 |
|  | **TNFα** | 0.391±0.021 | 0.482±0.006 | 0.395±0.027 | 0.411±0.022 | 0.437±0.015 | 0.379±0.016 |
| **Female** | **INFγ** | 1.058±0.057 | 1.399±0.126 | 1.090±0.043 | 1.016±0.013 | 1.091±0.094 | 1.144±0.039 |
|  | **IL1α** | 1.210±0.074 | 1.541±0.127 | 1.471±0.191 | 1.131±0.089 | 1.574±0.210 | 1.689±0.521 |
|  | **IL1β** | 4.589±0.189 | 4.870±0.286 | 4.718±0.163 | 4.278±0.230 | 3.911±0.146 | 4.305±0.088 |
|  | **IL6** | 2.913±0.301 | 4.256±0.117 | 2.730±0.361 | 2.268±0.431 | 2.483±0.251 | 2.570±0.227 |
|  | **IL10** | 0.932±0.043 | 0.935±0.124 | 1.028±0.158 | 0.887±0.066 | 0.884±0.140 | 0.965±0.078 |
|  | **TNFα** | 0.391±0.022 | 0.479±0.026 | 0.377±0.026 | 0.346±0.004 | 0.347±0.040 | 0.399±0.042 |

**Table S4:** Statistical power calculation (in percentage) of in vivo data.

| **Substantia nigra** | | *Injection* | *P-value* |  | *Genotype* | *P-value* |  | Sex | *P-value* |  |
| --- | --- | --- | --- | --- | --- | --- | --- | --- | --- | --- |
|  | **TH** | 100.00 | 0.00 | * | 100.00 | 0.00 | * | 30.17 | 0.15 |  |
|  | **Iba-1** | 100.00 | 0.00 | * | 100.00 | 0.00 | * | 88.88 | 0.00 | * |
|  | **CD86** | 100.00 | 0.00 | * | 99.97 | 0.00 | * | 36.50 | 0.11 |  |
|  | **Endpoints** | 76.44 | 0.01 | * | 93.33 | 0.00 | * | 100.00 | 0.00 | * |
|  | **Branch Length** | 94.31 | 0.00 | * | 92.98 | 0.00 | * | 100.00 | 0.00 | * |
|  | **INFγ** | 6.86 | 0.68 |  | 18.27 | 0.43 |  | 9.97 | 0.50 |  |
|  | **IL1α** | 5.71 | 0.80 |  | 19.95 | 0.39 |  | 72.93 | 0.01 | * |
|  | **IL1β** | 13.21 | 0.40 |  | 67.83 | 0.03 | * | 5.51 | 0.83 |  |
|  | **IL6** | 92.35 | 0.00 | * | 98.66 | 0.00 | * | 7.22 | 0.65 |  |
|  | **IL10** | 25.98 | 0.18 |  | 7.97 | 0.81 |  | 32.46 | 0.13 |  |
|  | **TNFα** | 44.47 | 0.07 |  | 64.34 | 0.03 | * | 6.97 | 0.67 |  |
|  | **CD68 area** | 100.00 | 0.00 | * | 100.00 | 0.00 | * | 7.30 | 0.66 |  |
|  | **Fractal dimension** | 19.46 | 0.27 |  | 62.06 | 0.04 | * | 7.95 | 0.61 |  |
|  | **Lacunarity** | 21.38 | 0.24 |  | 14.34 | 0.57 |  | 61.23 | 0.03 | * |
|  | **Density** | 99.99 | 0.00 | * | 94.49 | 0.00 | * | 11.85 | 0.45 |  |
|  | **Span ratio** | 99.69 | 0.00 | * | 65.08 | 0.03 | * | 7.99 | 0.61 |  |
|  | **Circularity** | 99.90 | 0.00 | * | 50.63 | 0.08 |  | 31.24 | 0.14 |  |
|  | **Maximum Span Across Hull** | 100.00 | 0.00 | * | 100.00 | 0.00 | * | 100.00 | 0.00 | * |
|  | **Area** | 100.00 | 0.00 | * | 100.00 | 0.00 | * | 27.46 | 0.17 |  |
|  | **Perimeter** | 100.00 | 0.00 | * | 100.00 | 0.00 | * | 11.17 | 0.47 |  |
|  | **Width of Bounding Rectangle** | 100.00 | 0.00 | * | 100.00 | 0.00 | * | 25.89 | 0.19 |  |
|  | **Height of bounding rectangle** | 100.00 | 0.00 | * | 100.00 | 0.00 | * | 51.48 | 0.05 |  |
|  | **Maximum Radius from Hull's centre of mass** | 100.00 | 0.00 | * | 100.00 | 0.00 | * | 16.14 | 0.33 |  |
|  | **Max/Min radii** | 99.04 | 0.00 | * | 69.90 | 0.02 | * | 10.25 | 0.50 |  |
|  | **CV for all Radii** | 96.29 | 0.00 | * | 22.62 | 0.36 |  | 37.26 | 0.10 |  |
|  | **Mean Radius** | 100.00 | 0.00 | * | 100.00 | 0.00 | * | 5.00 | 1.00 |  |
|  | **Diameter of bounding circle** | 100.00 | 0.00 | * | 100.00 | 0.00 | * | 11.61 | 0.45 |  |
|  | **Maximum radius from circle's centre** | 100.00 | 0.00 | * | 100.00 | 0.00 | * | 11.61 | 0.45 |  |
|  | **Max/min radii from Circle's Centre** | 92.38 | 0.00 | * | 53.05 | 0.07 |  | 15.75 | 0.34 |  |
|  | **CV for all Radii from circle's centre** | 81.77 | 0.00 | * | 59.40 | 0.05 |  | 73.29 | 0.01 |  |
|  | **Mean Radius from Circle's Centre** | 100.00 | 0.00 | * | 100.00 | 0.00 | * | 5.85 | 0.79 |  |
| **Striatum** | **Iba-1** | 99.20 | 0.00 | * | 99.54 | 0.00 | * | 45.19 | 0.07 |  |
|  | **CD86** | 100.00 | 0.00 | * | 100.00 | 0.00 | * | 5.95 | 0.77 |  |
|  | **Endpoints** | 99.90 | 0.00 | * | 91.68 | 0.00 | * | 88.13 | 0.00 | * |
|  | **Branch Length** | 98.47 | 0.00 | * | 89.33 | 0.00 | * | 47.18 | 0.06 |  |
|  | **INFγ** | 37.57 | 0.10 |  | 67.68 | 0.03 | * | 72.39 | 0.01 |  |
|  | **IL1α** | 34.26 | 0.12 |  | 33.42 | 0.19 |  | 25.05 | 0.19 |  |
|  | **IL1β** | 13.54 | 0.39 |  | 95.67 | 0.00 | * | 7.50 | 0.63 |  |
|  | **IL6** | 27.80 | 0.17 |  | 99.63 | 0.00 | * | 35.22 | 0.11 |  |
|  | **IL10** | 23.35 | 0.21 |  | 5.48 | 0.96 |  | 29.36 | 0.15 |  |
|  | **TNFα** | 41.60 | 0.08 |  | 79.35 | 0.01 | * | 41.94 | 0.08 |  |
|  | **CD68 area** | 100.00 | 0.00 | * | 100.00 | 0.00 | * | 6.07 | 0.76 |  |
|  | **Fractal dimension** | 7.05 | 0.67 |  | 41.34 | 0.14 |  | 100.00 | 0.00 | * |
|  | **Lacunarity** | 90.04 | 0.00 | * | 33.70 | 0.20 |  | 91.37 | 0.00 | * |
|  | **Density** | 100.00 | 0.00 | * | 100.00 | 0.00 | * | 99.81 | 0.00 | * |
|  | **Span ratio** | 12.67 | 0.42 |  | 85.04 | 0.00 | * | 49.88 | 0.05 |  |
|  | **Circularity** | 38.98 | 0.09 |  | 54.41 | 0.06 |  | 17.51 | 0.31 |  |
|  | **Maximum Span Across Hull** | 100.00 | 0.00 | * | 100.00 | 0.00 | * | 100.00 | 0.00 | * |
|  | **Area** | 100.00 | 0.00 | * | 100.00 | 0.00 | * | 5.19 | 0.90 |  |
|  | **Perimeter** | 100.00 | 0.00 | * | 100.00 | 0.00 | * | 5.40 | 0.85 |  |
|  | **Width of Bounding Rectangle** | 100.00 | 0.00 | * | 100.00 | 0.00 | * | 5.02 | 0.97 |  |
|  | **Height of bounding rectangle** | 100.00 | 0.00 | * | 100.00 | 0.00 | * | 77.20 | 0.01 | * |
|  | **Maximum Radius from Hull's centre of mass** | 100.00 | 0.00 | * | 100.00 | 0.00 | * | 14.48 | 0.37 |  |
|  | **Max/Min radii** | 15.27 | 0.35 |  | 55.81 | 0.06 |  | 44.84 | 0.07 |  |
|  | **CV for all Radii** | 27.53 | 0.17 |  | 57.55 | 0.05 |  | 38.87 | 0.09 |  |
|  | **Mean Radius** | 100.00 | 0.00 | * | 100.00 | 0.00 | * | 7.64 | 0.63 |  |
|  | **Diameter of bounding circle** | 100.00 | 0.00 | * | 100.00 | 0.00 | * | 12.47 | 0.43 |  |
|  | **Maximum radius from circle's centre** | 100.00 | 0.00 | * | 100.00 | 0.00 | * | 12.47 | 0.43 |  |
|  | **Max/min radii from Circle's Centre** | 17.36 | 0.31 |  | 60.41 | 0.04 | * | 33.56 | 0.12 |  |
|  | **CV for all Radii from circle's centre** | 30.56 | 0.15 |  | 70.88 | 0.02 | * | 92.99 | 0.00 | * |
|  | **Mean Radius from Circle's Centre** | 100.00 | 0.00 | * | 100.00 | 0.00 | * | 7.18 | 0.66 |  |
